# New targets of nascent lymphatic vessels in ocular diseases

**DOI:** 10.3389/fphys.2024.1374627

**Published:** 2024-03-11

**Authors:** Xuhui Wu, Yunkun Ma, Zhaochen Zhang, Tingting Hou, Yuxi He

**Affiliations:** ^1^ The Second Hospital of Jilin University, Changchun, Jilin, China; ^2^ Department of Orthopedics, The Second Hospital of Jilin University, Changchun, Jilin, China; ^3^ Department of Ophthalmology, The Second Hospital of Jilin University, Changchun, Jilin, China

**Keywords:** lymphangiogenesis, ocular diseases, corneal immune reaction, corneal (lymph)angiogenic privilege, corneal transplantation immunology

## Abstract

Recent advancements in the field of endothelial markers of lymphatic vessels and lymphangiogenic factors have shed light on the association between several ocular diseases and ocular nascent lymphatic vessels. The immune privilege of corneal tissue typically limits the formation of lymphatic vessels in a healthy eye. However, vessels in the eyes can potentially undergo lymphangiogenesis and be conditionally activated. It is evident that nascent lymphatic vessels in the eyes contribute to various ocular pathologies. Conversely, lymphatic vessels are present in the corneal limbus, ciliary body, lacrimal glands, optic nerve sheaths, and extraocular muscles, while a lymphatic vasculature-like system exists in the choroid, that can potentially cause several ocular pathologies. Moreover, numerous studies indicate that many ocular diseases can influence or activate nascent lymphatic vessels, ultimately affecting patient prognosis. By understanding the mechanisms underlying the onset, development, and regression of ocular nascent lymphatic vessels, as well as exploring related research on ocular diseases, this article aims to offer novel perspectives for the treatment of such conditions.

## 1 Background: ocular nascent lymphatic vessels

In healthy eyes, only the corneal limbus, conjunctiva, extraocular muscles, and lacrimal glands contain lymphatic vessels (Hou et al., 2021; Lee et al., 2021). However, the lymphatic vessel tissues such as the cornea and sclera are differentiated early in embryonic development and maintained by a balance of pro- and anti-lymphopoietic and immunomodulatory factors (Cursiefen et al., 2006a). A study by Collin in 1966 in which ink was injected into damaged rabbit vascularized cornea and allowed to flow into the ocular lymph nodes revealed the ultrastructure of the lymphatic vessels in the vascularized cornea and proposed the concept of lymphangiogenesis (Collin, 1966): the progression of new lymphatic vessels that develop based on existing lymphatic vessels or blood vessels through proliferation, migration, and differentiation in response to infection, inflammation, and chemical stimulation when the dynamic balance of lymphangiogenesis described above is disrupted and shifted in the direction of prolymphangiogenesis. Lymphangiogenesis plays dual roles in ocular diseases, and numerous studies have demonstrated that lymphangiogenesis can exacerbate dry eye disease, allergic disease, herpetic keratitis, and various other ocular diseases by promoting immune cell activation, increasing proinflammatory cytokines, and transporting antigen-presenting cells into regional lymph nodes to accelerate antigenic sensitization, which can ultimately result in corneal nerve damage and exacerbate clinical symptoms (Bock et al., 2013; Chennakesavalu et al., 2021; Lee et al., 2021). Moreover, recent research has demonstrated the physiological role of lymphangiogenesis in ocular inflammation; lymphangiogenesis is essential for termination of the physiological inflammatory response by facilitating the efflux of macrophages from the cornea, accelerating the resolution of corneal edema and opacity (Hos et al., 2017), maintaining avascularity in corneal tissue, and reducing the clinical symptoms of keratitis (Narimatsu et al., 2019). In recent years, there have been many discoveries regarding the role of lymphangiogenesis in the progression or amelioration of numerous ocular diseases. This article summarizes what is known about the onset, progression, regression of ocular nascent lymphatics and their roles in ocular diseases to create new treatment strategies.

## 2 The development process of nascent lymphatic vessels in the eye

The immune system, inherent to living organisms, is capable of recognizing non-self substances. The primary mechanism involves cells that present antigens to effector T cells, followed by the production of inflammatory cytokines or direct tissue damage by effector T cells, resulting in localized inflammation (Zarrin et al., 2021). Advanced organisms require more precise vision throughout their lifetimes; nevertheless, any minor change can lead to damage, while the inflammatory response caused by the immune system may be one of these changes. Therefore, organisms have evolved specific cells and molecules to form unique anatomical structures (such as blood–tissue barriers) that insulate the eye from inflammatory responses to prevent damage to vision, a phenomenon known as “immune privilege” (Streilein, 2003). Medawar et al. discovered this property in the cornea (Medawar, 1948). Additionally, the sclera also has an immune privilege (Atta et al., 2020). Barker et al. demonstrated that the cornea, anterior chamber, vitreous cavity, and subretinal space are immunoabsorbent (Barker and Billingham, 1977). Research has shown that this property is from interactions among several immune mechanisms (Niederkorn, 2006). Therefore, when the eye is stimulated by trauma or other factors, the nascent lymphatic vessels in this area, as well as immune cells and reactive substances, have an additional impact on this area and play a significant role in the development and progression of the disease.

**FIGURE 1:**
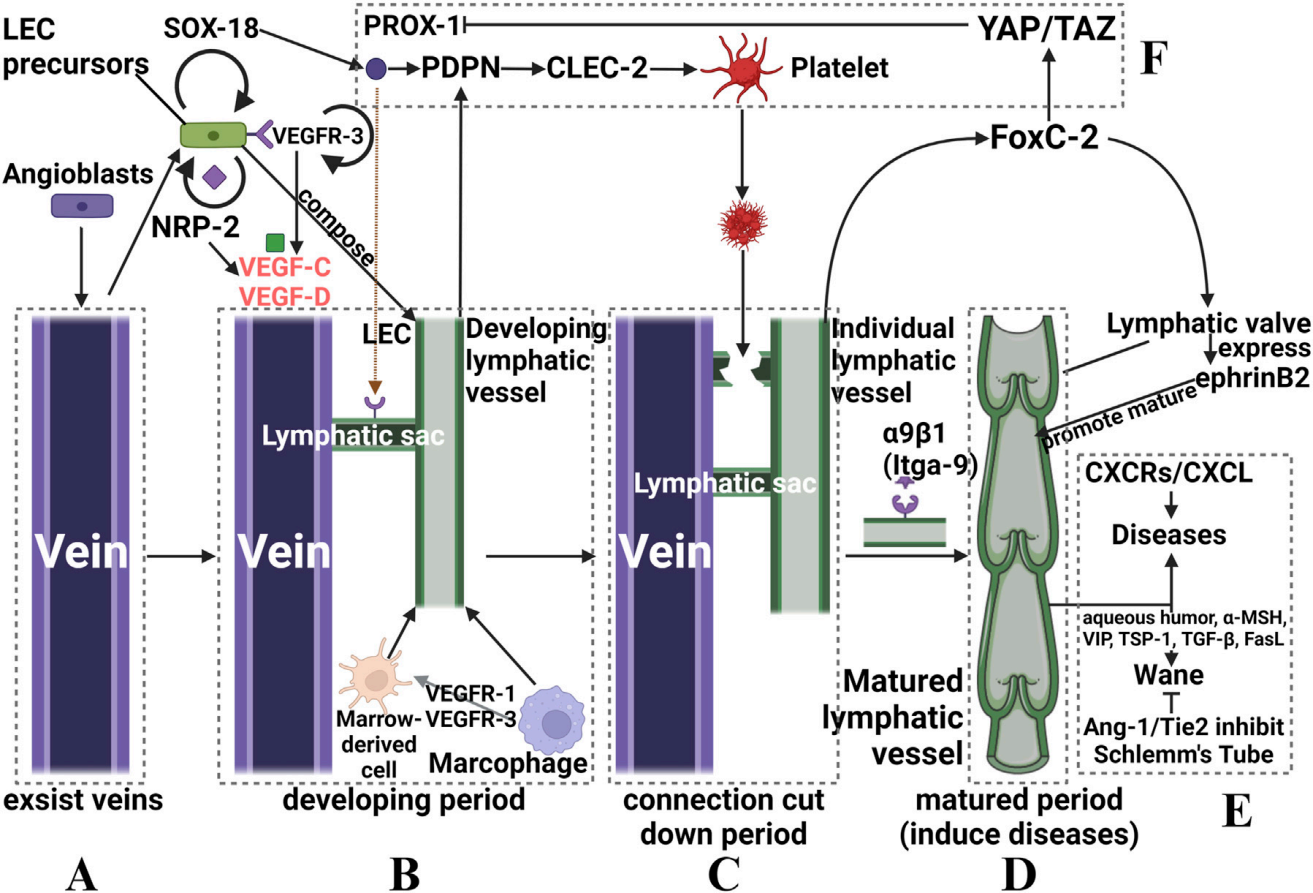
Schematic diagram of the occurrence and development of ocular nascent lymphatic vessels. **(A)**: angioblasts of existing veins stimulate LEC precursors. **(B)**: nascent lymphatic vessels formed that are connected to the vein. **(C)**: a subset of factors activate platelets to form thrombi, which can “cut down” the connection via localized necrosis. **(D)**: Itga-9 induces the formation of a lymphatic valve, which marks the birth of a mature nascent lymphatic vessel. **(E)**: pathogenesis or regression of nascent lymphatic vessels, and related regulatory factors. **(F)**: Other factors that assist in regulating lymphatic vessel formation. Although we split the whole process into four periods, they actually happened continuously.

In recent decades, the discovery of various lymphatic endothelial cell-specific markers, such as lymphatic vessel endothelial hyaluronan receptor 1 (LYVE-1), vascular endothelial growth factor receptor 3 (VEGFR-3), and homology box protein 1 (PROX-1) (Alitalo et al., 2005; Lee et al., 2021), has led to mechanistic studies of the occurrence, development, and regression of nascent lymphatic vessels. Notably, various stimuli to the eye, including inflammation, trauma, grafts, and infections, can disrupt the balance of lymphangiogenesis and promote the progression of lymphangiogenesis. This intricate process can be summarized in the following sequential steps:

1) First, preexisting endothelial cells differentiate into venous and arterial cells (Abdelfattah et al., 2016). Some venous endothelial cells express VEGFR-3, also called fms-related receptor tyrosine kinase 4 (flt-4), at high levels. Others begin to express LYVE-1, which is a precursor of lymphatic endothelial cells (LECs) (Tammela and Alitalo, 2010). LEC precursors also express neuropilin 2 (NRP-2), which does not directly activate downstream signaling factors but regulates VEGFR-3 expression levels involved in the regulation of lymphangiogenesis (Tang et al., 2016). (Figure 1A)
2) VEGFR3 and NRP-2 are expressed on the surface of LEC precursors and subsequently bind to vascular endothelial growth factor (VEGF)-C and VEGF-D (19), inducing the phosphorylation of two important survival signaling molecules, p42/p44 mitogen-activated protein kinase (MAPK) and serine/threonine kinase (AKT), which promote the migration, proliferation and survival of LECs (Mäkinen et al., 2001; Lee et al., 2021). Some cells also express VEGFR2 and bind to VEGF-A, thus playing a partial role in lymphangiogenesis and lymphatic vessel function maintenance (Alitalo et al., 2005). Furthermore, macrophages in vessels secrete VEGF-C and VEGF-D while also expressing VEGFR-1 and VEGFR-3, which mediate myeloid cell (such as macrophages) chemotaxis and thus perpetuate lymphangiogenesis (Cursiefen et al., 2004). Additionally, myeloid cells can directly integrate into nascent lymphatic vessels and transdifferentiate into LECs (Hong and Detmar, 2003) (Figure 1B).
3) The precursors of LECs express SRY-box transcription Factor 18 (SOX-18), which is upstream of PROX-1 and induces these cells to express PROX-1, migrate and differentiate into lymphatic vessels (François et al., 2008). Some of the PROX-1-positive cells adhere to the exterior of the vein, where they form “pro-lymphoid clusters (PLCs)" that later swell to form a lymphatic capsule while remaining connected to the vein (Uhrin et al., 2010).
  PROX-1 activates the expression of podoplanin (PDPN) in LECs (Pan et al., 2014), and PDPN activates calcium-dependent lectin-like receptor 2 (CLEC-2) in platelets, triggering platelet activation and/or aggregation through downstream signaling, which involves spleen tyrosine kinase (Syk) and SRC homology 2 (SH2) domain-containing leukocyte phosphoprotein of 76 kDa (SLP-76) activation. Finally, the lymphatics and the veins from which they originate are ultimately separated to form distinct lymphatics (Figure 1F).
4) At this time, LECs begin to express downstream factors of VEGFR-3, such as the Forkhead transcription factor C2 (FoxC2) (Abdelfattah et al., 2016), which positively inhibits factors that negatively regulate the expression of PROX-1, such as Yes-associated protein (YAP) and transcriptional coactivator with a PDZ-binding motif (TAZ) (Cha et al., 2020); concurrently, LECs express the integrin α9β1 (Itga-9) (Altiok et al., 2015), and valvular LECs highly express ephrinB2 (28); all these factors collectively mediate lymphatic flap formation and ultimately the formation of a mature lymphatic vessel (Figure 1C); subsequently, the mature vessels enter the final stage and induce disease.
5) In contrast to the development of nascent lymphatic vessels, their regression is poorly understood. LECs are among the few differentiated cells that require continuous gene expression to maintain their phenotypic characteristics (Johnson et al., 2008). Therefore, downregulation of VEGFR-3 and PROX-1 expression can induce reprogramming of LECs to dedifferentiate toward vascular endothelial cells, leading to regression of lymphatic vessels (Ma et al., 2021). In addition, Shi et al. demonstrated that atrial fluid could induce lymphatic regression in normal and pathological conditions and linked immunomodulatory factors such as α-melanocyte-stimulating hormone (a-MSH), vasoactive intestinal peptide (VIP), thrombospondin (TSP-1), transforming growth factor β (TGF-β), and Fas ligand (FasL) to lymphatic regression (Shi et al., 2020). However, the underlying mechanisms require further investigation (Figure 1E). Furthermore, other important factors also play a crucial role in the process, as described below.

**FIGURE 2:**
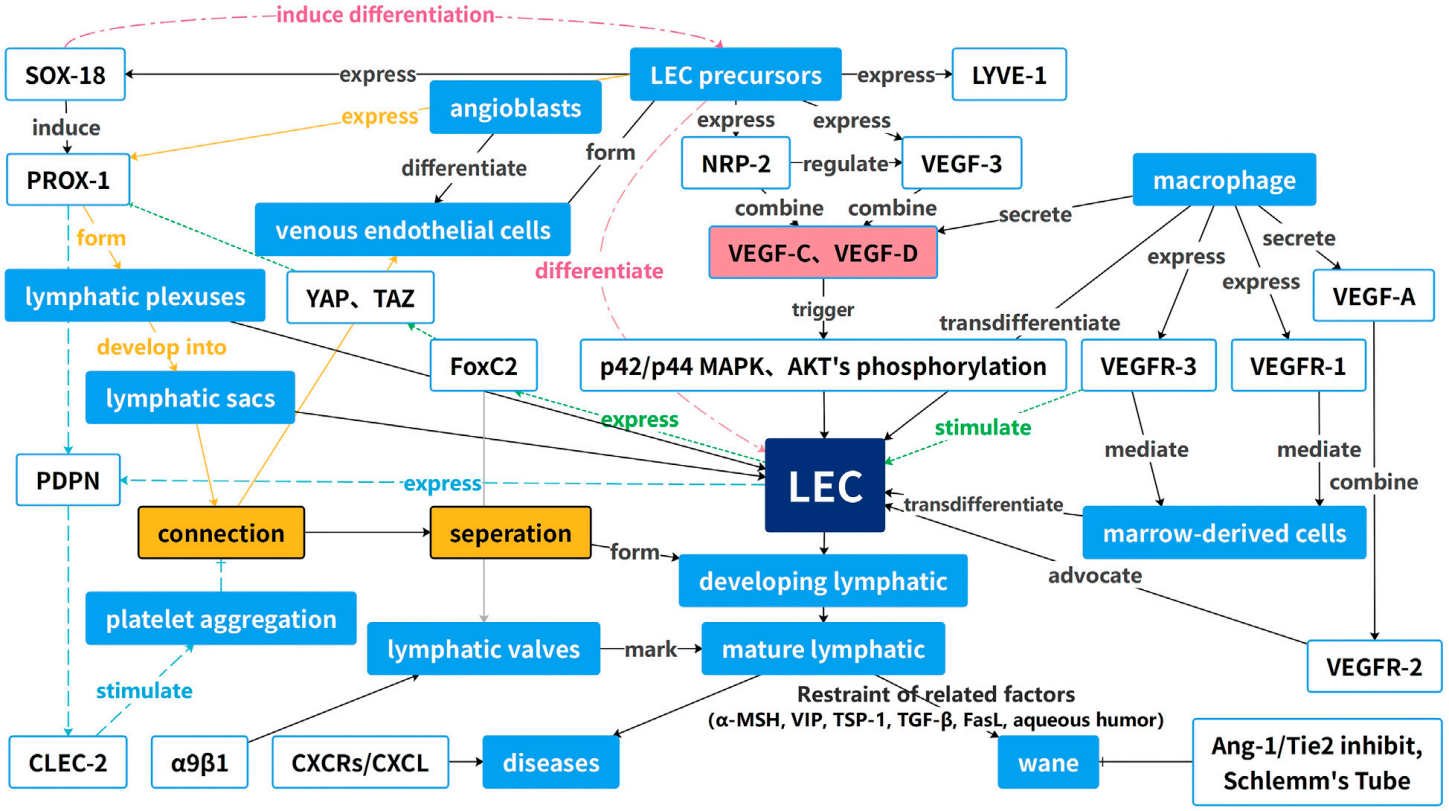
Guide map of the occurrence and development of ocular neoplastic vessels. This diagram reveals the interactions of these factors with receptors and how these factors increase or decrease the formation of LECs and nascent lymphatic vessels.

Numerous essential regulators of the process described above affect the progression of lymphangiogenesis in a pathological state. Among them, VEGF is the most important regulator of lymphangiogenesis; the VEGF-C/VEGFR-3 signaling pathway is the primary driver of lymphangiogenesis, while the VEGF-A/VEGFR-2 signaling pathway plays a secondary role (Kubo et al., 2002; Patnam et al., 2023). VEGF-C/-D is predominantly expressed by macrophages and corneal epithelial cells and binds to VEGFR-3 on LECs. This interaction activates intracellular signaling pathways, including PI3K-Akt and MAPK-ERK, leading to increased lymphatic proliferation, migration, and survival (Kuonqui et al., 2023). VEGF-A also promotes corneal lymphangiogenesis by stimulating macrophages to secrete VEGF-C and VEGF-D (35), and other factors, such as Notch, which may indirectly influence angiogenesis by regulating the VEGF/VEGFR signaling pathway (Zhu et al., 2021). Chemokine receptors (CXCRs) and their ligands (CXCLs) can stimulate nascent lymphatic vessel growth and intercommunication. VEGF-C induces the expression of CXCR4, which promotes lymphangiogenesis independently of the VEGF-C/VEGFR-3 signaling pathway. CXCL4 is expressed in the lymphatic endothelium, and CXCL12 is expressed in the adjacent tissues of nascent lymphatic vessels (Zhuo et al., 2012). In ocular nascent vessels, the CXCR3-CXCL10, CCR7-CCL19, and CCR7-CCL21 chemokine axes also play crucial roles (Gao et al., 2017; Wang et al., 2021). As an important angiogenic signaling pathway, Ang-1/Tie2 signaling is also essential for ocular lymphatic vessel neogenesis. Angiopoietin does not play a decisive role in lymphangiogenesis but rather in lymphatic vessel maturation. The effects of angiopoietin on lymphatic vessels may be mediated by influencing LEC interactions with surrounding smooth muscle cells, as suggested by previous research (Gale et al., 2002). In contrast to other factors, the Ang-1/Tie2 pathway is essential for the maintenance of Schlemm’s canal and is subject to severe regression and increased intraocular pressure in the absence of Tie2 and Ang1/Ang2, which may contribute to glaucoma (Kim et al., 2017). All these factors and tissues form a large network to induce or regulate the formation of nascent lymphatic vessels, as shown in Figure 2.

## 3 Distribution and characteristics of lymphatic vessels in ocular structures

**FIGURE 3:**
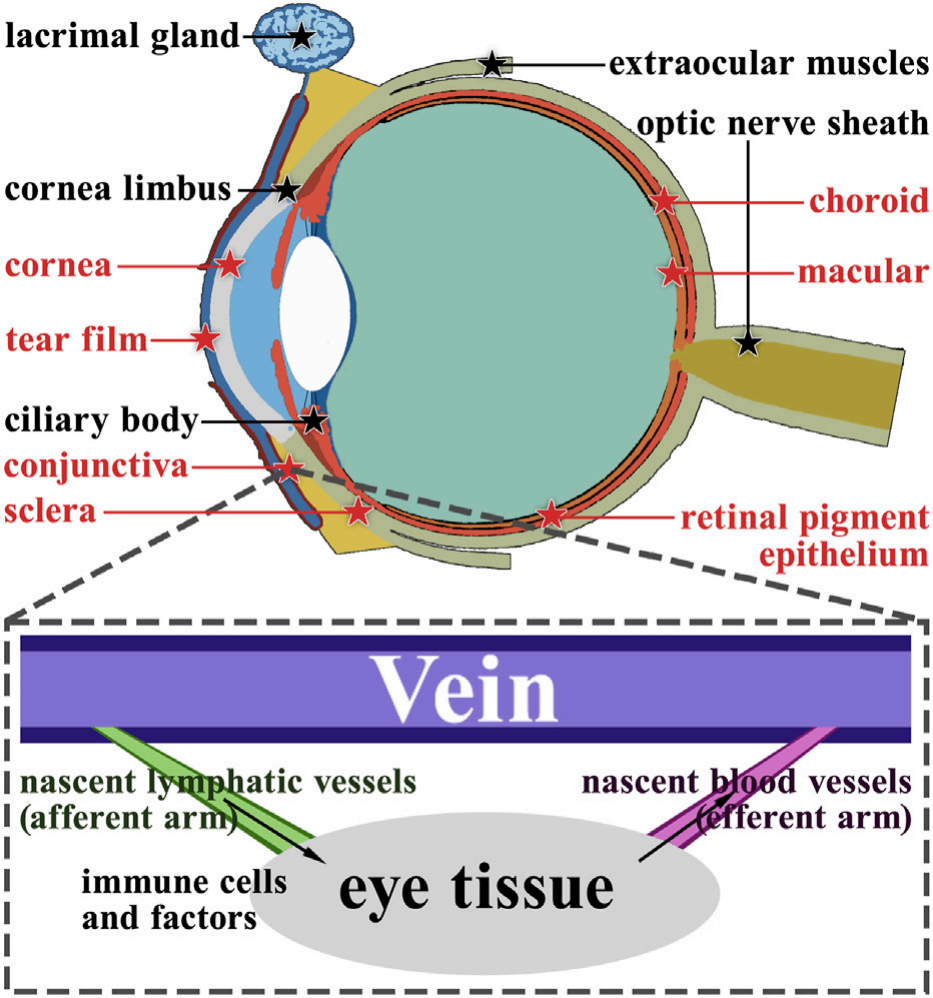
Anatomy of the ocular lymphatic system. The structures marked with black pentagrams represent the structures that have lymphatic vessels naturally. On the other hand, the structures marked with red pentagrams do not have lymphatic vessels naturally. Using the conjunctiva as an example, we demonstrate the role of nascent lymphatic vessels and blood vessels in ocular diseases.

Research has shown that functional lymphatic vessels are present in multiple anatomical structures within the eye (Figure 3). These structures include the corneal limbus, ciliary body, lacrimal gland, optic nerve sheath, and extraocular muscles. Additionally, the choroid is reported to have a lymphatic-like structure (Hou et al., 2021; Lee et al., 2021), The choroid does not have direct access to light as often as other tissues of the body, so they have their lymphatics. These lymphatic vessels and cells have defense, surveillance, and self-stabilization functions, which are the basic features of the immune system (Kaplan, 2007). Moreover, structures with no lymphatics will be seriously influenced by nascent lymphatics, which means that they will develop different lesions (Barker and Billingham, 1977).

## 4 Advances in nascent lymphatics in multiple ocular diseases

It is widely recognized that angiogenesis plays a major role in many pathological processes, as corneal lymphangiogenesis is hardly visible with common imaging and is difficult to observe (Doh et al., 2018). However, it is now believed that lymphangiogenesis is equally as significant as angiogenesis. Cursirfen et al. demonstrated that lymphangiogenesis and angiogenesis may be triggered by the same pathophysiological factors, grow in parallel, but subside at different times (Cursiefen et al., 2006b). Ecoiffier et al.‘s research also reported blood and lymphatic vessels parallelly occur in the cornea in a murine model of suture placement, though blood vessels lose their original nasal polarity during inflammation, lymphatic vessels maintain it (Ecoiffier et al., 2010). Research has shown that multiple anatomical structures within the eye possess functional lymphatic vessels, whereas other ocular structures do not (Barker and Billingham, 1977). However, nascent lymphatics are not involved in other ocular structures when various disorders occur, including corneal graft rejection, alkali burns, infectious keratitis, dry eye, pterygium, allergic eye disease, conjunctival melanoma, uvea-related disease, retina-related disease, and glaucoma. This article introduces and summarizes these diseases in the order of anatomical structure from ocular surface to cornea, choroid, and intraocular diseases.

### 4.1 Diseases related to the ocular surface structures

The ocular surface structures include the eyelids, conjunctiva, lacrimal apparatus, extraocular muscles, and orbits, of which the lacrimal apparatus and extraocular muscles are known to contain lymphatics normally, while the other structures do not contain lymphatics in the ordinary physiologic state (Kaplan, 2007; Hou et al., 2021; Lee et al., 2021).

#### 4.1.1 Dry eye disease

##### 4.1.1.1 Definition of dry eye disease

Dry eye disease (DED) is an ocular surface disorder characterized by significant irritation, visual impairment, and pain. It is caused by hyperosmolarity of the weeping film or damage/inflammation of the ocular surface (Ocul Surf, 2007). Previously, DED was interpreted merely as a manifestation of tear film deficiency. However, recent advancements have revealed DED to be a chronic and persistent inflammatory condition activated by a variety of internal and external factors (Chennakesavalu et al., 2021).

##### 4.1.1.2 DED led by lymphangiogenesis

There is evidence that the pathogenesis of DED involves the activation of both the innate and adaptive immune systems (Messmer, 2015). Lymphatic neovascularization mediates the activation and motility of immune cells on the corneal surface, and VEGF-C increases the size of lymphatic vessels. Goyal et al. were the first to demonstrate that in a murine model of DED, corneal lymphatic neovascularization occurred but was not accompanied by neovascularization (Goyal et al., 2010). Furthermore, the frequency of CD11 antigen-like family member B (CD11b) (+)/LYVE-1 (+) cells was elevated in localized lymph nodes, indicating a connection between nascent lymphatic vessels in DED and the activation of adaptive immune responses in lymph nodes. In other words, the discovery of factors such as LYVE-1 and VEGF-C in the DED disease model corroborates the formation of neolymphatic vessels in this disease, and the fact that these events are not accompanied by neovascularization reflects the direct and dominant role of neolymphatic vessels in this process.

However, recent research has also investigated additional lymphatic vasculature-related mechanisms in DED. In contrast to previous findings that Dll4/Notch predominantly regulates VEGF-C/VEGFR3 expression (Min et al., 2016), Dll4/Notch signaling regulates VEGF-D/VEGFR3 expression to inhibit lymphangiogenesis. Activation of hypoxia-inducible factor-1alpha (HIF-1α) similarly upregulates Notch expression and interacts with Notch-activated VEGFR3 to form new lymphatic vessels. However, the present study demonstrated that HIF-1α knockdown inhibited only Notch signaling and lymphangiogenesis. However, the relationship between HIF-1α and the Notch pathway in ocular nascent lymphatic vessels requires further analysis. The above two studies suggest that Notch signaling plays a complex and opposing regulatory role in the development of DED, but since the available data for the study of this phenomenon are still incomplete, additional experimental support is needed to explore the specific regulatory mechanisms and associated equilibrium of VEGF. Additionally, Wang et al. demonstrated the association between dendritic cell migration to nascent lymphatic vessels in DED and the chemokine axes CCR7-CCL19 and CCR7-CCL21. The authors also suggested that the CCR7-CCL19 chemokine axis may significantly impact dendritic cell migration in DED more than the CCR7-CCL21 chemokine axis (Wang et al., 2021).

From these studies, it is evident that the mechanism underlying the development and regulation of lymphangiogenesis in DED patients is still somewhat unclear. In addition to the factors identified in this study, such as VEGF and LYVE, additional factors and their interactions require further experimental and clinical data support.

##### 4.1.1.3 Signal blocking and corresponding treatments

Later studies confirmed the importance of nascent lymphatic vessels in the pathology of DED, and a subsequent study by Goyal et al. demonstrated that the VEGF-C antibody significantly reduced the caliber and size of lymphatic vessels in DED mice, resulting in decreased activation and recruitment of CD11 b+ cells, thus considerably improving DED symptoms (Goyal et al., 2012). IL-17 inhibition similarly inhibited lymphangiogenesis in a murine model of DED, revealing a mechanism by which Th17 cells and IL-17 induce DED lymphangiogenesis (Chauhan et al., 2011). In addition, research in the murine model showed topical IL-1 blockade (Okanobo et al., 2012) and the external application of epigallocatechin gallate (EGCG) can be effective in the treatment of DED by reducing corneal IL-1B expression and lymphatic neovascularization (Lee et al., 2011). Therefore, effective containment of several factors, such as VEGF-C, IL-17, IL-1, and IL-1B, effectively reduces or prevents lymphangiogenesis and is effective at inhibiting the development of DED.

#### 4.1.2 Pterygium

Pterygium is an abnormal wing-like growth of epithelial and fibrovascular tissue from the scleral limbus that invades the cornea centripetally, impairing vision and causing inflammation, the pathogenesis of which is primarily due to prolonged ultraviolet light exposure, and, occasionally, clinically involves invasive inflammation with neovascularization (Cárdenas-Cantú et al., 2016). Cimpean et al. examined pterygium by immunohistochemistry and discovered that lymphangiectasia density was greater in pterygium conjunctiva than in normal conjunctiva and that D2-40 (a LEC-specific marker)-positive LECs actively proliferate in pterygium, suggesting that lymphangiogenesis is activated during pterygium development (Cimpean et al., 2011). Both lymphatic vessel density and lumen diameter correlate with pterygium size, and nascent lymphatic vessels may contribute to pterygium growth, as demonstrated by Liu (Liu et al., 2012). In some studies, the lymphatic microvessel density was also used as a significant predictor of the latency to pterygium recurrence (Lin et al., 2013).

As in other diseases caused by nascent lymphatic vessels, the VEGF-C/VEGFR-3 pathway also plays a vital role in lymphangiogenesis in pterygium (Fukuhara et al., 2013), and ultraviolet light irradiation upregulates the expression of tumor necrosis factor α (TNF-α), which mediates an increase in the expression of VEGF-C in pterygium and promotes lymphangiogenesis (Dong et al., 2016). The expression of VEGF-A was significantly greater than that of VEGF-C, suggesting that neovascularization may be more significant in pterygium vessels than in nascent lymphatic vessels (Martín-López et al., 2019). The exact roles of neovascularization and lymphatic neovascularization in pterygium require additional study.

The above-related studies indicate that UV-induced upregulation of VEGF-C plays an important role in the pathogenesis of pterygium. Although the expression of VEGF-A indicates that neovascularization may play a greater role in this process, the exact contribution ratio of both to the pathogenesis of pterygium still needs further investigation. Determining their significance in treating this disease and improving targeted therapy are of practical significance.

#### 4.1.3 Allergic eye disease

Allergic eye disease is mediated by specific mediators (such as Th2 lymphocytes, mast cells, and eosinophils) and is characterized by intense inflammation of the conjunctiva and cornea, sensitivity to light, and complications that may lead to permanent impairment of vision (Dahlmann-Noor et al., 2021). In this disease, the upregulation of VEGFR-3 causes the expression of nascent lymphatic vessels. Additionally, it increases the reactivity of lymphocytes, as indicated by Th2 and IgE expression; thus, inhibiting VEGFR-3 may reduce the incidence of allergic reactions.

Lee et al. established a mouse model of allergic ophthalmopathy using ovalbumin exposure. They demonstrated that significant corneal nascent vessels were present in allergic ophthalmopathy and that VEGFR inhibition by Axitinib, a selective second-generation tyrosine kinase inhibitor of all VEGFRs. Significantly decreased the Th2 immune response and IgE levels and reduced the number of neoplastic vessels (Lee et al., 2015). Lou et al. recently demonstrated that VEGFR-3 inhibition modulates the activation and development of macrophages, Th2 cells, B cells, and mast cells in the conjunctival allergy-induced Th3 immune response and that VEGFR-3 is essential for the treatment of allergic ophthalmopathy (Lou et al., 2022). In addition, NF-κB is hypothesized to be associated with lymphangiogenesis and is implicated in the pathogenesis of allergic ocular disease. However, the exact underlying mechanism has not yet been elucidated (Lan et al., 2012).

Overall, these studies demonstrated that during the pathological changes observed in allergic ophthalmopathy, nascent lymphatics appear in ocular tissues, and experiments on the attenuation of allergic reactions by inhibiting VEGFR confirmed the close connection between nascent lymphatics and ocular allergic diseases; thus, inhibiting the process of nascent lymphatic vessel generation can reduce the development and progression of allergic ophthalmopathies to a certain extent.

#### 4.1.4 Conjunctival melanoma

Conjunctival melanoma accounts for 1%–2% of all ocular melanomas and is characterized by variable pigmentation, typically located on the nasal or temporal conjunctiva (Vasalaki et al., 2017). In this disease, conjunctival melanoma cells diffusely express VEGF-C, VEGF-D, and VEGFR-3, the most common of which are expressed at the tumor margin, and lymphangiogenesis-related factors, such as CXCL12, CXCR4, CCL21, and CCR7, are most strongly expressed at the margins of invasive tumors (Briceño et al., 2016), where they induce neovascularization. With the emergence of large numbers of tumor-associated neovascular structures in the periphery and inside of tumors, the number of peri-tumor neovascularizations decreases with increasing distance from the tumor boundaries, supporting the hypothesis of tumor-induced neovascularization (Zimmermann et al., 2009). There may be a connection between lymphangiogenesis and tumor progression. Heindl et al. reported that lymphangiogenesis occurs early in the development of precancerous intraepithelial lesions in conjunctival melanoma patients, and the increase in lymphangiogenesis coincides with the progression of precancerous lesions to invasive conjunctival melanoma. The intratumoral density of neolymphatic vessels correlates with the risk of local recurrence in patients with precancerous lesions. However, the relationship between lymphangiogenesis and the malignant transformation of tumors has not yet been established (Heindl et al., 2010a). In addition, they discovered that the development of precancerous lesions in conjunctival squamous cell carcinoma is accompanied by the growth of nascent lymphatic vessels, which has prognostic value for both metastasis and local recurrence (Heindl et al., 2010a). In 2015, Schlereth et al. successfully established a mouse model of conjunctival melanoma that can be used to investigate the role of different immune cells, cytokines, or growth factors in tumor-associated nascent lymphatic vessels and conjunctival melanoma, leading to the development of new antitumor therapies based on immunomodulatory and antilymphangiogenic strategies (Schlereth et al., 2015).

Research on conjunctival melanoma has shown that nascent lymphatic vessels tend to form at the edges of such tumors, reflecting the degree of tumor progression. Therefore, dynamic monitoring or identification of the levels of VEGF and CXCL in different parts of the patient’s conjunctiva or the density or number of nascent lymphatic vessels is quite important, as these levels can reflect the risk, degree, and location of recurrence of conjunctival melanoma to some extent. Hence, the focus of research on nascent lymphatic vessels in this disease is on prognostication and recurrence judgment, which differs from the other diseases mentioned in this article.

### 4.2 Cornea-related diseases

The cornea is a transparent connective tissue, the secretions on its surface and itself constitute the physical and biological barrier of the eye and are also part of the refractive structure of the eye (DelMonte and Kim, 2011). Due to its refractive function and location, it is the first structure encountered by light on its pathway, devoid of lymphatic vessels and blood vessels. Therefore, diseases occurring at the cornea are affected not only by external stimuli but also by the formation of nascent lymphatic vessels.

The diseases known to occur in the cornea associated with lymphatic vessels include transplant rejection, alkali burns, and infectious keratitis, and the nascent lymphatics in these diseases all act as immune afferent arms (Hou et al., 2021). Cornea-related diseases often cause serious eye health and vision problems; therefore, we will introduce the relevant content in detail below.

#### 4.2.1 Corneal transplant rejection

##### 4.2.1.1 Mechanism of corneal transplant rejection

To understand the importance of the corneal nascent lymphatic vessels in the development of disease, their significance in the cornea needs to be addressed first. Due to the absence of blood vessels and lymphatic vessels into which immune cells can enter, the cornea has the characteristic immune privilege of being unresponsive to foreign transplanted tissues and other antigenic stimuli (Iwamoto and Smelser, 1965). Since the eye itself does not contain these tissues, not does not have any preexisting defense mechanisms against the corresponding reaction when it occurs. (Bock et al., 2013; Chennakesavalu et al., 2021; Lee et al., 2021). Overall, these findings indicate that under normal circumstances, there are no immune cells in the human lymphatic system in the cornea; therefore, there is no immune response between the human body and corneal tissue. This also results in the cornea naturally lacking resistance to such immune responses. Therefore, once an immune response occurs in this region, it is easier than in other tissues that have a lymphatic system. Corneal allografts have been effective for more than half a century, but graft rejection is still possible. During graft rejection, nascent lymphatics and blood vessels constitute the afferent and efferent arms of the immune reflex arc; nascent blood vessels in particular act as the efferent arm by facilitating the migration and infiltration of immune cells into the transplanted cornea (Lee et al., 2021). Several studies have highlighted that neovascularization is a dynamic process controlled by equilibrium mechanisms, such as the expression of soluble VEGF receptor 1 (sVEGFR-1) by the cornea, which inhibits the trapping of endogenous VEGF-A by neutralizing antibodies (Ambati et al., 2007). However, factors such as RNA interference or gene disruption mediated by Cre–lox mice can disrupt this mechanism to enable neovascularization (Ambati et al., 2006); In contrast, nascent lymphatics act as immune afferent arms, facilitating the entry of immune cells into the cornea and transporting antigens to the lymph nodes, thereby accelerating the sensitization process (Hou et al., 2021). Recent research has emphasized the crucial role of nascent lymphatics in corneal graft rejection, with Dietrich et al. demonstrating their significance in the murine model compared to neovascularization (Dietrich et al., 2010). Moreover, the presence of a lymphatic vasculature system has been identified as an indication of a high-risk state during transplantation (Regenfuss et al., 2008). In summary, compared to neovascularization, the formation of nascent lymphatic vessels as an afferent arm can promote the entry of immune cells into the cornea and trigger an immune response, accelerating sensitization and leading to graft rejection, which highlights the importance of studying lymphangiogenesis.

It has been demonstrated that there is a risk of immune rejection during corneal transplantation. This process can be summarized as follows: ① The generated veins in the cornea can express VEGFR-3 and bind VEGF-C and VEGF-D with NRP-2 on the LEC precursors (Vaahtomeri et al., 2017). ② Meanwhile, LEC precursors were stimulated to promote the expression of Itga-9 to induce the formation of lymphatic flaps that generate independent corneal lymphatic vessels (Altiok et al., 2015). ③ After the formation of lymphatic flaps, which signalsthe formation of the mature corneal lymphatic vessels, these vessels are connected to the recipient’s lymphatic system as are other lymphatic vessels of the body, this process was previously shown in Figure 1D before. The recipient’s immune cells subsequently enter the cornea through these nascent lymphatic vessels, defying the immune privilege of the cornea. ④ Because of the deficiencies of existing defense reactions against these cells and factors, recipient lymphocytes subsequently attack the foreign cornea, thereby initiating an immune rejection response, which is similar to the defense reaction to external stimuli such as bacteria, viruses, or fungi. Immune cells recognize the cells and tissues of foreign corneas as foreign materials and express T cells to activate cytotoxic reactions to protect the body. ⑤ These immune cells increase in number and induce inflammation in the whole area of the eye. As the inflammation becomes more severe enough to reach the threshold, the tissues in these regions will be destroyed, and this process is called the immune rejection response. It has been demonstrated that inhibiting neovascularization and lymphangiogenesis during corneal transplantation significantly reduces graft rejection (Bock et al., 2013).

Tammela et al. discovered that lymphangiogenesis must be initiated and conducted based on angiogenesis, a previously described process (Tammela and Alitalo, 2010). Hos et al. summarized the indications for various types of corneal transplants (modern lamellar transplants (DALK, DSAEK, and DMEK) *versus* conventional penetrating corneal transplantation) and the risk of rejection in light of the sequential occurrences of angiogenesis and lymphangiogenesis as well as the influence of related factors (Hos et al., 2019). Lamellar keratoplasty, despite significantly reducing the rate of graft rejection, is still not satisfactory in patients with severe inflammation and pathological angiogenesis and lymphangiogenesis. Moreover, perioperative allergic conjunctivitis can also accelerate lymphatic vessel neovascularization, resulting in more severe graft rejection (Flynn et al., 2011). Although these technologies have achieved certain success in treating these diseases, they still face challenges, such as a high occurrence rate of immune rejection reactions, potential unclarified biological processes, and individual patient variability, which could affect the success rate of these technologies. Therefore, the regulatory mechanisms and influencing factors of lymphangiogenesis discussed in this article provide a new perspective for further reducing the risk of transplant rejection and improving the overall success rate of transplantation.

##### 4.2.1.2 Treatment of corneal transplant rejection

###### 4.2.1.2.1 Signaling pathway blockade

While topical steroid drops remain the standard treatment for corneal graft rejection, scientists have successfully developed a number of new therapies based on the mechanism of ocular lymphangiogenesis to inhibit lymphangiogenesis, thereby decreasing the rate of corneal graft rejection and specifically inhibiting the VEGF system or Itga-9, thereby ameliorating clinical symptoms which will be introduced below. Anti-VEGF therapies have been evaluated in the clinic and partially implemented (Bock et al., 2008; Ferrari et al., 2013). Drugs such as soluble VEGFRs, anti-VEGFR3 antibodies, VEGFR tyrosine kinase inhibitors, and integrin-blocking peptides are currently undergoing preclinical testing (Albuquerque et al., 2009; Detry et al., 2013; Hou et al., 2021). The VEGFR1/R2 trap neutralizes VEGF-A and effectively inhibits corneal lymphatic neovascularization, thereby enhancing corneal graft survival in patients at high risk (Bachmann et al., 2008). One of the VEGFR1/R2 traps, aflibercept, has been shown to inhibit neovascularization (lymphatic vessels) more effectively than VEGF-C antibodies and sVEGFR-3, resulting in enhanced long-term survival in high-grade corneal grafts. Aflibercept is approved for use in clinical practice (Dohlman et al., 2015). Recent studies additionally indicate that preincubation of corneal donor tissue with a VEGFR1R2 cytokine trap has the remarkable capacity to substantially enhance the survival of high-risk corneal transplants, thereby paving the way for novel therapeutic approaches in high-risk corneal transplantation (Zhang et al., 2022a).

According to the previously described mechanism of lymphangiogenesis, the maturation of lymphatic vessels requires the formation of lymphatic flaps facilitated by Itga-9. In addition to VEGF-related therapies, the inhibition of Itga-9 with specific antibodies represents a promising strategy for effectively suppressing lymphatic valve formation, impeding the maturation of nascent lymphatics, and perturbing immune responses, thereby facilitating allograft survival (Kang et al., 2016). Angiopoietin-2 is also critically involved in lymphangiogenesis processes *in vivo* and *in vitro*. Corneal lymphangiogenesis response is almost eliminated in Ang-2 knockout mice (Yuen et al., 2014). L1-10, an Ang-2-specific inhibitor, significantly inhibited lymphangiogenesis but promoted angiogenesis, providing a strong rationale for the exploitation of anti-Ang-2 treatment in the prevention and treatment of transplant rejection (Yan et al., 2012). In a related study between fully mismatched C57BL/6 (donor) and BALB/c (recipient) mice, Ang-2 blockade via neutralizing antibody was found to significantly inhibit lymphangiogenesis and graft rejection. Moreover, the morphology of macrophages is affected, and central corneal thickening is inhibited in transplanted corneas, providing a novel strategy for the treatment of corneal transplant rejection (Zhang et al., 2017). Furthermore, NRP-2 blockade (Tang et al., 2010), insulin receptor substrate 101 (IRS-1) blockade (Hos et al., 2011), TSP-1 blockade (Cursiefen et al., 2011), and matrix metalloproteinase (MMP) blockade (Maruyama et al., 2014) have shown potential for inhibiting lymphangiogenesis, thereby promoting the survival of corneal grafts. However, further research is necessary before these approaches can be employed in clinical settings. Notably, Yu et al. reported an intriguing observation regarding chemokine receptor-2 (ACKR-2) in the murine model of corneal transplant sutures. While previous studies have indicated that ACKR-2 might interfere with the immune response and reduce lymphangiogenesis in inflammation-related contexts, it did not impact the rate of corneal graft rejection (Yu et al., 2018). This phenomenon suggested that ACKR-2 may play different roles in different phases of lymphangiogenesis, warranting further investigation.

###### 4.2.1.2.2 Inhibition of lymphocyte activation

In addition, since macrophages can express VEGF-C/-D, VEGFR-3, and other related receptors and factors, the induction of corneal lymphangiogenesis by macrophages plays a crucial role in corneal transplant rejection (Cursiefen et al., 2004). However, these medications have not yet been approved for clinical use. Zhu et al. demonstrated that ex vivo-induced myeloid-derived suppressor cells block angiogenesis and lymphangiogenesis and inhibit the activation of effector T cells through the Jak3/STAT5 signaling pathway via the inducible nitric oxide synthase (iNOS) pathway, thus improving corneal graft survival (Zhu et al., 2021). Subconjunctival injection of dimethyl fumarate in rats and mice and modulation of the intracellular thiol oxidation status also reduces macrophage infiltration, modulate lymphangiogenesis, and ultimately decrease corneal graft rejection rates (Fukumoto et al., 2010; Yu et al., 2021).

###### 4.2.1.2.3 Other emerging treatments

Several physical therapies have recently been found to be effective in suture‐induced inflammatory murine models at eliminating corneal lymphatics and decreasing vascularization, in addition to the abovementioned studies on the underlying mechanisms and associated therapies. Among these therapies, ultraviolet A-based corneal collagen crosslinking (CXL) has shown promising results. By stimulating apoptosis in vascular endothelial cells, CXL leads to a notable regression of nascent lymphatics and vascularization without affecting nonvascular endothelial cells in the cornea, iris, or lens. Consequently, this approach significantly enhances the long-term survival rate of corneal grafts following transplantation (Hou et al., 2018). Another noteworthy clinical therapy, fine-needle diathermy (FND), has been demonstrated to effectively reduce the occurrence of allograft rejection subsequent to high-risk penetrating corneal transplantation (Ravichandran and Natarajan, 2022). Furthermore, photodynamic therapy (PDT) has been shown to abrogate corneal lymphangiogenesis and angiogenesis in a time-dependent manner and to promote the survival of high-risk corneal allografts, possibly through a mechanism in which the release of highly reactive cytotoxic oxygen radicals from verteporfin activated by laser energy leads to endothelial cell damage and ultimately to lymphatic and vascular occlusion and ablation (Hou et al., 2017). Mesenchymal stem cells (MSCs) have also shown promising potential for preventing transplant rejection because of their decreased expression of vascular endothelial growth factor C (VEGF-C) and increased expression of Fas (Kang et al., 2023).

Overall, these studies suggest that innovations in methods or improvements in strategies for blocking and regulating different aspects of lymphangiogenesis can significantly reduce the risk of immune rejection in corneal transplantation, which is the anticipated goal of this article.

#### 4.2.2 Alkali burns

Corneal alkali burns usually result in permanent visual impairment, and studies have shown that angiogenesis, lymphangiogenesis and inflammatory reactions that develop after alkali burns result in ocular disease with the worst prognosis after corneal transplantation (Yamada et al., 2003). The presence of corneal nascent vessels correlates significantly with the inflammatory response in a rat model of corneal alkali burns, emphasizing the crucial function of nascent lymphatic vessels as immune afferent arms after alkali burns (Yan et al., 2010). Alkali burns have also been used as a modeling tool to induce inflammatory lymphangiogenesis. Zhang et al.'s experiments in the murine model of incision injury, alkali burn, suture placement, low-risk keratoplasty, and high-risk keratoplasty revealed that corneal alkali burns produced fewer nascent lymphatic vessels than suture and graft burn wounds but significantly more nascent lymphatic vessels than incision burns (Zhang et al., 2022b).

The mechanism of alkali burns involves distinct pathways of lymphangiogenesis regulatory factors compared to those involved in corneal graft rejection. One notable distinction is the upregulation of the CXCR system (Li et al., 2022), which potentially leads to a reduction in the expression of VEGF-A and VEGF-C. Additionally, as the inflammatory response subsides, the secretion of VEGF-C decreases (Shen et al., 2014). Notably, during the process of lymphatic flap formation, the involvement of EphB4 receptor tyrosine kinases and their transmembrane adrenergic B2 ligands assumes a crucial role under both conditions (Lyons et al., 2021). These differences highlight the unique mechanisms and factors involved in lymphangiogenesis following alkali burns, differing from corneal graft rejection.

Lymphangiogenesis in corneal alkali burns may be closely related to the inflammatory index, and when the inflammatory response tends to subside, VEGF-C secretion decreases and induces nascent lymphangiogenesis before vascularization; moreover corneal transplantation at this time dramatically reduces the probability of rejection (Yan et al., 2010); however, the relationship between the inflammatory response and lymphangiogenesis requires further investigation. Overall, the prognosis for patients with alkali burns is poor, and the upregulation of CXCR with inflammatory responses reflects the difference in the role of nascent lymphatic vessels in this condition compared to their role in other corneal diseases.

Multiple other factors also play crucial roles in the development of alkali burns. Several of them have been described to some extent. Previously, it was believed that the CXCR3 chemokine was expressed in newly formed blood vessels because it binds to CXCL10, where it controls the expression of VEGF-A and C and indirectly affects effectors such as MMP13 and Serpine1, thereby inhibiting the migration of endothelial cells and the formation of new lumina (Gao et al., 2017). Therefore, inhibiting CXCR3 and thus attenuating VEGF expression while controlling the transition of endothelial cells to the nascent lymphatic vessel wall could also be a means of alleviating lymphangiogenesis and mitigating alkali burns; however, it is important to note that CXCR3 expression had no effect on lymphangiogenesis according to the study conducted by Li et al. This finding may suggest that CXCR3 participates in distinct mechanisms during corneal angiogenesis and lymphangiogenesis (Li et al., 2022), which need further investigation. EphB4 receptor tyrosine kinase and its transmembrane epinephrine B2 ligand-mediated signaling play critical roles in the formation and maintenance of flaps in corneal neoplastic vessels, and disruption of ephrinB2-EphB4 signaling interferes with the afferent arm of the immune reflex arc by affecting lymphatic flap formation and leukocyte-lymphoid interactions (Katsuta et al., 2013) so that lymphangiogenesis is inhibited, thereby reducing alkali burn injury in the eye. Furthermore, as shown by some research *in vitro* corneal tissue cells, human dermal lymphatic endothelial cells (HDLEC) and alkali burned mouse models, Hydrocinnamoyl-L-valylpyrrolidine (AS-1) inhibits lymphangiogenesis via the inhibition of extracellular signal-regulated kinase phosphorylation (Liu et al., 2021), Lanepitant through the antagonism of neurokinin-1 (NK-1) (Bignami et al., 2014), and microRNA miR-466 and Prox1 siRNA targeting of PROX-1 (Rho et al., 2015; Seo et al., 2015) can all inhibit lymphangiogenesis, providing a novel therapeutic approach for alkali burn-induced lymphangiogenesis. In conclusion, the various treatment methods used reflect the complexity of the factors that cause corneal alkali burns, and their interactions are more diverse, hence revealing new pathways for targeted treatments based on individual differences.

#### 4.2.3 Infectious keratitis

Infectious keratitis is the primary infectious cause of blindness worldwide (Farooq and Shukla, 2012), and corneal infections are frequently accompanied by the appearance of lymphatic neovascularization and neovascularization (Rowe et al., 2013). Herpes stromal keratitis (HSK) is a prevalent form of infectious keratitis caused by herpes simplex virus 1 (HSV-1) (Wang et al., 2020). HSK is characterized by immunoinflammatory corneal lesions and vision impairment caused by neovascularization of lymphatic vessels (Ahmad and Patel, 2024).

Moreover, HSV-1-induced lymphangiogenesis was more strongly dependent on VEGF-A/VEGFR-2 signaling than on VEGF-C and D/VEGFR-3 signaling in combination with the two corneal diseases mentioned above (Ambati et al., 2006; Yun et al., 2020). In addition, HSV-1-induced neovascularization was not associated with macrophages and was less effective at antigen presentation than VEGF-C- or -D-induced lymphangiography (Wuest and Carr, 2010). Contrary to previous assumptions, CD8^+^ T cells, rather than CD4^+^ T cells, suppressed viral replication in the cornea of HSK patients but upregulated VEGF-C expression and promoted lymphangiogenesis. Conrady et al. suggested that VEGF-A initiates lymphangiogenesis and that VEGF-C promotes angiogenesis after HSV infection (Conrady et al., 2012). It has also been demonstrated that fibroblast growth factor 2 (FGF-2) and interleukin-6 (IL-6) play central roles in the induction and maintenance of corneal neoplastic vessels (Gurung et al., 2018).

During lymphangiogenesis following HSV-1 infection of the cornea, type 1 interferon and its intact receptor system help to block viral replication and inhibit lymphangiogenesis. Bryant-Hudson et al. demonstrated that, compared with that in wild-type mice, lymphangiogenesis was accelerated in transgenic mice lacking the type 1 interferon receptor (Bryant-Hudson et al., 2013). The abovementioned ACKR-2 indirectly restricts corneal angiogenesis in HSK through its ability to scavenge inflammatory chemokines and macrophages but only in mild inflammation and without impacting viral replication (Yu et al., 2022).

The above research indicates that infectious keratitis caused by HSV-1 has different characteristics in terms of the underlying mechanism and influencing factors than the other two diseases, suggesting that treatments for this disease should not be confused with those for other types of corneal diseases. This finding suggests that there are more treatment options, discovered or undiscovered, to be explored in this field, as well as the development and validation of cross-treatment schemes for different diseases, laying a foundation for new directions for treating this disease.

In addition to HSK, other infectious keratoids are associated with lymphatic neoplasia to varying degrees. Qin et al. discovered that Resolvins D1 (RvD1) in diabetic *Aspergillus fumigatus* keratitis effectively decreased IL-8 and IL-6 levels, the fungal load, and ROS production by blocking the MAPK-NF-κB pathway, along with promoting vascularization and lymphangiogenesis (Qin et al., 2022). Another study on bacterial keratitis caused by *Pseudomonas aeruginosa* revealed that late bacterial keratitis activates VEGF-C/VEGFR-3 signaling and macrophages, inducing corneal lymphangiogenesis. Intriguingly, their study showed that nascent lymphatics in advanced stages of bacterial keratitis help reduce edema and inflammation-induced corneal clouding, revealing the positive impact of nascent lymphatics in human pathophysiological processes and suggesting that induced lymphangiogenesis, despite being the cause of numerous diseases, could be a novel therapeutic option for bacterial keratitis (Narimatsu et al., 2019).

These findings, in addition to providing additional insight into infectious keratitis caused by other pathogens, also highlight the beneficial role of nascent lymphatic vessels in alleviating certain pathological processes in humans. The suggestion that promoting lymphangiogenesis can treat bacterial keratitis challenges the traditional view that lymphangiogenesis is merely a pathological factor. Given that this type of research is still in its early stages, future studies may focus on whether nascent lymphatic vessels are also beneficial for other diseases in humans.

### 4.3 Uveal-related diseases

The uvea is divided into three parts: the iris, the ciliary body and the choroid. There are no lymphatic vessels, and nascent lymphatic vessels may have extraocular extensions, thus facilitating the migration of inflammation or tumors. This section provides a relevant summary of tumors (Refaian et al., 2015).

#### 4.3.1 Ciliary body melanoma

Ciliary body melanoma is extremely rare. Primarily because of its concealed location, it usually only becomes apparent in more advanced stages. The most common symptom of this disease is blurred vision. Hematogenous metastasis of ciliary body melanomas is rapid. It promotes the formation of nascent lymphatic vessels due to the continuous contraction of the ciliary muscle and the high vascularity of the ciliary body (Costache et al., 2013). Studies by Heindl et al. have shown that nascent lymphatic vessels are present in ciliary body malignant melanomas with extraocular extensions but not in melanomas without extrascleral extensions (Heindl et al., 2009; Heindl et al., 2010b). They also suggested that peritumoral nascent lymphatics are a prognostic factor in extraocular extended ciliary body melanoma (Heindl et al., 2010b). However, Khan et al. noted that ciliary body nascent lymphatics can develop without extraocular extension (Khan et al., 2013); therefore, whether lymphangiogenesis can be used as a prognostic indicator for this tumor and the specific mechanism of lymphangiogenesis in ciliary body melanomas are still topics that need further exploration. Nonetheless, tumor-associated lymphangiogenesis is a risk factor for this tumor type.

However, studies on uveal-related diseases indicate that the role of nascent lymphatic vessels in these diseases is still unclear, necessitating further research and data support. This point will be mentioned later in the paper.

### 4.4 Interocular tissue diseases

#### 4.4.1 Glaucoma

**FIGURE 4:**
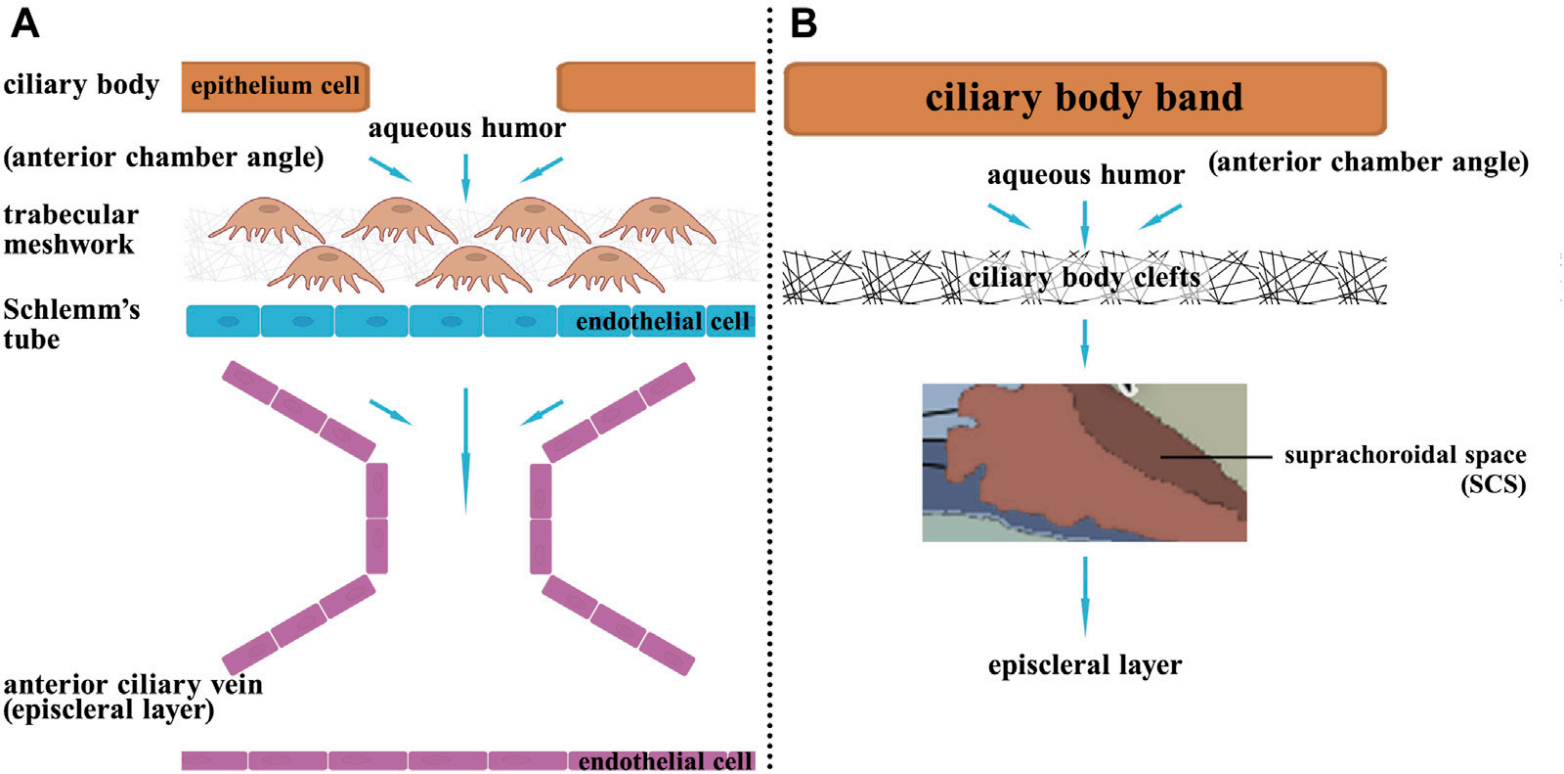
The two pathways of aqueous humor circulation. Aqueous humor is generated from the ciliary processes, then it flows from the posterior chamber through the pupil to the anterior chamber. Subsequently, there are two outflow pathways: **(A)** Trabecular meshwork pathway: 80% of the aqueous humor exits through this pathway. The aqueous humor enters Schlemm’s canal through the trabecular meshwork in the anterior chamber angle, then drains into the episcleral veins via Schlemm’s canal. **(B)** Uveoscleral pathway: 20% of the aqueous humor exits through this pathway. The aqueous humor passes through the ciliary body band and then enters the ciliary body clefts in the anterior chamber angle, then enters the suprachoroidal space, and finally exits the eye by passing through the sclera.

Glaucoma is the second leading cause of blindness worldwide. It is a chronic, degenerative disease of the optic nerve (Jayaram et al., 2023). Several known risk factors for glaucoma include elevated intraocular pressure, age, genetic background, thinning of the cornea and vascular disorders, and one of the most important is increased intraocular pressure, which is caused by the resistance of fluid flow through the trabecular meshwork (Weinreb et al., 2014). This process is primarily caused by impaired drainage of the aqueous humor, ultimately resulting in irreversible injury to retinal ganglion cells (Almasieh et al., 2012). The conventional trabecular outflow pathway and the unconventional uveoscleral outflow pathway are the two routes of atrial fluid discharge (Figure 4).

In this disease, the formation of nascent lymphatic vessels is linked to both conventional and nonconventional pathways. The conventional pathway leads to the formation of Schlemm’s vessels, which are similar to lymphatic structures and are regulated by the VEGF system. In comparison, the unconventional pathway involves lymphatic drainage (Aspelund et al., 2014; Oliver et al., 2020). Therefore, when VEGF-related factors are downregulated, the formation of these structures is inhibited, leading to impaired aqueous drainage and subsequent glaucoma. The detailed mechanisms of each of the two pathways are outlined below.

Approximately 80% of the body’s atrial fluid passes through the trabecular meshwork into Schlemm’s canal, where it then drains into the external scleral vein, and the absence, underdevelopment, or acquired alteration of the Schlemm’s canal is associated with primary glaucoma (Oliver et al., 2020). The Schlemm’s canal, a lumen with molecular and morphological characteristics of blood and lymphatic vessels, was recently found to originate in the choroidal veins, and the epithelium was found to exhibit lymphatic endothelial cell characteristics by upregulating Prox1 (129). The Schlemm canal possesses remarkable structural and functional similarities with lymphatic vessels, creating a blood-free blind tube that transports atrial fluid and antigen-presenting cells into the venous circulation. Its molecular regulatory mechanisms are also similar to those of the lymphatic system. Aspelund et al. demonstrated that a lack of VEGF-C inhibits Schlemm’s canal development in murine and zebrafish models as well as in human eye tissue (Yücel et al., 2009). And that the development and maintenance of Schlemm’s canal and atrial fluid efflux also require PROX-1 and Tie2 signaling, which could lead to new therapies for the treatment of glaucoma (Park et al., 2014; Kim et al., 2017).

Moreover, approximately 20% of atrial fluid can also be absorbed from the anterior chamber through the interstitial space of the ciliary muscle across the sclera by the orbital vasculature or enter the choroid and exit through the vortex vein, after which the fluid returns to the venous circulation (Johnson et al., 2017). Yucel et al.‘s experiments in live sheep indicate that the aqueous fluid exiting the anterior chamber is partially directed towards lymph nodes responsible for draining the head and neck area, instead of directly communicating with the bloodstream (Yücel et al., 2009). Tam et al. demonstrated that latanoprost, a prostaglandin F2 analog, increased lymphatic drainage via the uveoscleral pathway by 400% in murine (Tam et al., 2013), indicating that its efficacy is primarily related to lymphatic vessels, but further studies are needed to determine the role of nascent lymphatic vessels in this process. In addition, a recent study revealed that bone morphogenetic protein 9 (BMP9), despite inhibiting lymphatic vessel maturation and valve formation, has no effect on the Schlemm’s canal or intraocular microscopic lymphatic vessels and has no effect on intraocular pressure (IOP), rendering it unsuitable for use as a novel therapeutic target for glaucoma (Subileau et al., 2020).

In conclusion, since glaucoma is one of the leading causes of blindness worldwide, there is a great demand for new and effective treatment options. VEGF factors promote the formation of Schlemm’s canal and lymphatic vessels, helping to lower intraocular pressure by draining aqueous humor, thereby serving as a potential improvement measure for treating this disease. As mentioned earlier, this is also the beneficial role of lymphangiogenesis in human disease, but how to ensure that other eye structures are not damaged during the treatment of glaucoma remains a topic for further research.

### 4.5 Diseases with unconfirmed presence but potential risk of nascent lymphatic vessels

Uveitis refers to the inflammation of a group of uveal tissues (iris, ciliary body, and choroid) and is frequently caused by ocular hypertension, retinal ischemia, or macular edema (Krishna et al., 2017). Uveitis is mainly autoimmune mediated inflammation (also occurs with infection and trauma), where nascent lymphatic vessels can act as immune afferent arms of the immune reflex arc (Hos et al., 2008). The levels of LYVE-1 and PDPN detected in the diseased tissues of patients with uveitis due to Behcet’s disease were significantly different from those observed in the corresponding tissues of healthy subjects (Özgürtaş et al., 2022). Another study also revealed significantly elevated levels of VEGFR-1 and sVEGFR-3 in patients with Behcet’s uveitis (Sertoglu et al., 2022), all of which suggested that the disease may be related to lymphangiogenesis and that factors such as PDPN may provide new options for treating Behcet’s uveitis.

Choroidal melanoma, a subtype of uveal melanoma, is a common intraocular malignancy. Despite the presence of VEGF-C and its receptor Flt-4, choroidal melanoma was previously believed to not contain nascent lymphatic vessels, consistent with its lack of lymphatic metastasis and reliance on the hematogenous metastasis pathway (Clarijs et al., 2001). However, in rare cases, lymphatic vessels spread regionally, and it is impossible to confirm the presence or absence of nascent lymphatic vessels in choroidal melanoma. Nevertheless, “atypical” lymphatic markers may be expressed in the eyes (van Beek et al., 2019), and additional research is needed to confirm the role of nascent lymphatic vessels and their associated markers in choroidal melanoma.

Age-related macular degeneration (AMD) is the primary cause of irreversible central vision loss. It is a degenerative disease of the macula. AMD alters retinal pigment epithelium and choroid function AMD is classified pathophysiologically as dry AMD or wet AMD. Wet AMD, also known as neovascular AMD, causes more severe vision loss in patients than dry AMD (Sarkar et al., 2022). The role of neovascularization in AMD is well understood, but the function of nascent lymphatic vessels in the pathogenesis of AMD still needs to be fully understood. Ikeda et al. demonstrated that VEGF-C and VEGF-D were expressed in ocular specimens from patients with AMD and that VEGF-C and VEGF-D expression was regulated by factors such as hypoxia and cellular matrix adherence but did not emphasize their regulation of lymphangiogenesis (Ikeda et al., 2006). Nakao et al. also showed that VEGF-C expression was upregulated in AMD but did not result in typical lymphangiogenesis (Nakao et al., 2013); similar to what was observed in choroidal melanoma mentioned above, Cabral proposed that this result may involve the interaction between VEGF-A and VEGF-C (Cabral et al., 2018). Additional research is required to determine the function of nascent lymphatic vessels in AMD.

### 4.6 Future direction

At present, the mechanisms related to the formation of eye diseases are still in the developing stage, and there are few studies on nascent lymphatics. At present, more and more people need to undergo diversified treatments for eye diseases to achieve better therapeutic effects. Various studies summarized in this paper point out the importance of nascent lymphatics in several eye diseases. Given that the current research on the relationship between lymphangiogenesis and ocular diseases is more detailed, we believe that there will be more in-depth research on the pathogenic mechanism, occurrence and development of lymphangiogenesis as well as therapeutic targets. More new therapeutic strategies and targeted drugs will be found based on the characteristics of lymphangiogenesis (Park et al., 2014; Kim et al., 2017; Narimatsu et al., 2019). Moreover, the current research mainly focuses on animal models, and the relevant clinical trials are few, so the clinical validation of existing therapeutic methods may also be the object of attention in the future. Studying the cross-talk between lymphangiogenesis and other biological processes, such as angiogenesis, fibrosis, and metabolism will also help to elucidate the complex interplay between different systems and processes in health and disease (Wang et al., 2023).

Advancing imaging technologies to visualize lymphatic vessels and lymphangiogenesis *in vivo* with higher resolution and specificity is also an emerging research direction. Improved imaging techniques will facilitate the study of lymphatic biology in real-time and in the context of the whole organism (Doh et al., 2018).

## 5 Summary

In this review, we comprehensively examined the mechanisms underlying the formation, progression, and regression of neoplastic vessels, as well as their implications for ocular diseases. While the majority of related studies have focused primarily on neovascularization, this review provides a comprehensive overview of the mechanisms and disease associations of neoplastic vessels, highlighting relevant loci that could serve as targets for clinical treatment and drug discovery. By gaining a deeper understanding of these mechanisms, we can enhance therapeutic efficacy and alleviate symptoms in affected patients. However, it is essential to note that the current research on neovascularization and lymphatic vessels predominantly focuses only on neovascularization, and further investigations are required to elucidate the specific mechanisms and roles of neoplastic vessels in different ocular diseases. Moreover, studies translating these theories into clinical applications or involving relevant animal models are lacking. Additionally, the association of neoplastic vessels with other diseases and the precise underlying mechanisms remain urgent areas for exploration.

## Figures and Tables

**Table:** 

**ACKR2**	Atypical Chemokine Receptor 2
**AKT**	Serine/threonine kinase
**AMD**	Age-related macular degeneration
**a-MSH**	α-melanocyte-stimulating hormone
**AS-1**	Hydrocinnamoyl-L-valylpyrrolidine
**BMP9**	Bone morphogenetic protein 9
**CD11b**	CD11 antigen-like family member B
**CLEC-2**	Calcium-dependent lectin-like receptor 2
**CXCL**	Chemokine receptors ligands
**CXCRs**	Chemokine receptors
**DALK**	Deep Anterior Lamellar Keratoplasty
**DED**	Dry eye disease
**DMEK**	Descemet’s membrane endothelial keratoplasty
**DSAEK**	Descemet’s stripping automated endothelial keratoplasty
**EGCG**	Epigallocatechin gallate
**FasL**	Fas ligand
**FGF-2**	Fibroblast growth factor 2
**flt-4**	Fms related receptor tyrosine kinase 4
**FND**	Fine-Needle Diathermy
**FoxC2**	Forkhead transcription factors C2
**HDLEC**	Human dermal lymphatic endothelial cells
**HIF-1α**	Hypoxia-inducible factor-1alpha
**HSK**	Herpes stromal keratitis
**HSV-1**	Herpes simplex virus
**IL-6**	Interleukin-6
**iNOS**	Inducible Nitric Oxide Synthase
**IOP**	Intraocular pressure
**IRS-1**	Insulin receptor substrate 101
**Itga-9**	Integrin α9β1
**LECs**	Lymphatic endothelial cells
**LYVE-1**	Lymphatic vessel endothelial hyaluronan receptor 1
**MAPK**	Mitogen-activated protein kinase
**MMP**	Matrix metalloproteinase
**MSCs**	Mesenchymal stem cells
**NK-1**	Neurokinin-1
**NRP-2**	Neuropilin 2
**PDPN**	Podoplanin
**PDT**	Photodynamic therapy
**PLCs**	Pro-lymphoid clusters
**PROX-1**	Homology box protein 1
**RvD1**	Resolvins D1
**SH2**	SRC homology 2
**SLP-76**	SH2 domain-containing leukocyte phosphoprotein of 76 kDa
**SOX-18**	SRY-box transcription factor 18
**sVEGFR-1**	Soluble VEGF receptor 1
**Syk**	Spleen tyrosine kinase
**TAZ**	PDZ-binding motif
**TGF-β**	Transforming growth factor β
**TNF-α**	Tumor necrosis factor α
**TSP-1**	Thrombospondin
**VEGF**	Vascular endothelial growth factor
**VEGFR**	Vascular endothelial growth factor receptor
**VIP**	Vasoactive intestinal peptide
**YAP**	Yes-associated protein

## References

[B1] Abdelfattah N. S. Amgad M. Zayed A. A. Hussein H. Abd El-Baky N. (2016). Molecular underpinnings of corneal angiogenesis: advances over the past decade. Int. J. Ophthalmol. 9 (5), 768–779. 10.18240/ijo.2016.05.24 27275438 PMC4886880

[B2] Ahmad B. Patel B. C. (2024). Herpes simplex keratitis. Treasure Island (FL): StatPearls Publishing. 31424862

[B3] Albuquerque R. J. C. Hayashi T. Cho W. G. Kleinman M. E. Dridi S. Takeda A. (2009). Alternatively spliced vascular endothelial growth factor receptor-2 is an essential endogenous inhibitor of lymphatic vessel growth. Nat. Med. 15 (9), 1023–1030. 10.1038/nm.2018 19668192 PMC2882165

[B4] Alitalo K. Tammela T. Petrova T. V. (2005). Lymphangiogenesis in development and human disease. Nature 438 (7070), 946–953. 10.1038/nature04480 16355212

[B5] Almasieh M. Wilson A. M. Morquette B. Cueva Vargas J. L. Di Polo A. (2012). The molecular basis of retinal ganglion cell death in glaucoma. Prog. Retin Eye Res. 31 (2), 152–181. 10.1016/j.preteyeres.2011.11.002 22155051

[B6] Altiok E. Ecoiffier T. Sessa R. Yuen D. Grimaldo S. Tran C. (2015). Integrin alpha-9 mediates lymphatic valve formation in corneal lymphangiogenesis. Invest. Ophthalmol. Vis. Sci. 56 (11), 6313–6319. 10.1167/iovs.15-17509 26431485 PMC4911102

[B7] Ambati B. K. Nozaki M. Singh N. Takeda A. Jani P. D. Suthar T. (2006). Corneal avascularity is due to soluble VEGF receptor-1. Nature 443 (7114), 993–997. 10.1038/nature05249 17051153 PMC2656128

[B8] Ambati B. K. Patterson E. Jani P. Jenkins C. Higgins E. Singh N. (2007). Soluble vascular endothelial growth factor receptor-1 contributes to the corneal antiangiogenic barrier. Br. J. Ophthalmol. 91 (4), 505–508. 10.1136/bjo.2006.107417 17151056 PMC1994740

[B9] Aspelund A. Tammela T. Antila S. Nurmi H. Leppänen V. M. Zarkada G. (2014). The Schlemm’s canal is a VEGF-C/VEGFR-3-responsive lymphatic-like vessel. J. Clin. Invest. 124 (9), 3975–3986. 10.1172/JCI75395 25061878 PMC4153703

[B10] Atta G. Tempfer H. Kaser-Eichberger A. Guo Y. Schroedl F. Traweger A. (2020). The lymphangiogenic and hemangiogenic privilege of the human sclera. Ann. Anat. 230, 151485. 10.1016/j.aanat.2020.151485 32120002

[B11] Bachmann B. O. Bock F. Wiegand S. J. Maruyama K. Dana M. R. Kruse F. E. (2008). Promotion of graft survival by vascular endothelial growth factor a neutralization after high-risk corneal transplantation. Arch. Ophthalmol. 126 (1), 71–77. 10.1001/archopht.126.1.71 18195221

[B12] Barker C. F. Billingham R. E. (1977). Immunologically privileged sites. Adv. Immunol. 25, 1–54. 345773

[B13] Bignami F. Giacomini C. Lorusso A. Aramini A. Rama P. Ferrari G. (2014). NK1 receptor antagonists as a new treatment for corneal neovascularization. Invest. Ophthalmol. Vis. Sci. 55 (10), 6783–6794. 10.1167/iovs.14-14553 25228541

[B14] Bock F. König Y. Kruse F. Baier M. Cursiefen C. (2008). Bevacizumab (Avastin) eye drops inhibit corneal neovascularization. Graefes Arch. Clin. Exp. Ophthalmol. 246 (2), 281–284. 10.1007/s00417-007-0684-4 17934753

[B15] Bock F. Maruyama K. Regenfuss B. Hos D. Steven P. Heindl L. M. (2013). Novel anti(lymph)angiogenic treatment strategies for corneal and ocular surface diseases. Prog. Retin. Eye Res. 34, 89–124. 10.1016/j.preteyeres.2013.01.001 23348581

[B16] Briceño C. A. Elner V. M. Demirci H. (2016). Lymphangiogenic and chemotactic factors in conjunctival melanoma. Ophthalmic Plast. Reconstr. Surg. 32 (6), 428–433. 10.1097/IOP.0000000000000567 26460963

[B17] Bryant-Hudson K. M. Chucair-Elliott A. J. Conrady C. D. Cohen A. Zheng M. Carr D. J. J. (2013). HSV-1 targets lymphatic vessels in the eye and draining lymph node of mice leading to edema in the absence of a functional type I interferon response. Am. J. Pathol. 183 (4), 1233–1242. 10.1016/j.ajpath.2013.06.014 23911821 PMC3791868

[B18] Cabral T. Lima L. H. Mello L. G. M. Polido J. Correa É. P. Oshima A. (2018). Bevacizumab injection in patients with neovascular age-related macular degeneration increases angiogenic biomarkers. Ophthalmol. Retina 2 (1), 31–37. 10.1016/j.oret.2017.04.004 29376143 PMC5783314

[B19] Cárdenas-Cantú E. Zavala J. Valenzuela J. Valdez-García J. E. (2016). Molecular basis of pterygium development. Semin. Ophthalmol. 31 (6), 567–583. 10.3109/08820538.2014.971822 25415268

[B20] Cha B. Ho Y. C. Geng X. Mahamud M. R. Chen L. Kim Y. (2020). YAP and TAZ maintain PROX1 expression in the developing lymphatic and lymphovenous valves in response to VEGF-C signaling. Development 147 (23), dev195453. 10.1242/dev.195453 33060128 PMC7758626

[B21] Chauhan S. K. Jin Y. Goyal S. Lee H. S. Fuchsluger T. A. Lee H. K. (2011). A novel pro-lymphangiogenic function for Th17/IL-17. Blood 118 (17), 4630–4634. 10.1182/blood-2011-01-332049 21908425 PMC3208279

[B22] Chennakesavalu M. Somala S. R. R. Dommaraju S. R. Peesapati M. P. Guo K. Rosenblatt M. I. (2021). Corneal lymphangiogenesis as a potential target in dry eye disease - a systematic review. Surv. Ophthalmol. 66 (6), 960–976. 10.1016/j.survophthal.2021.03.007 33811911 PMC9991079

[B23] Cimpean A. M. Poenaru Sava M. Raica M. Ribatti D. (2011). Preliminary evidence of the presence of lymphatic vessels immunoreactive for D2-40 and Prox-1 in human pterygium. Oncol. Rep. 26 (5), 1111–1113. 10.3892/or.2011.1342 21667034

[B24] Clarijs R. Schalkwijk L. Ruiter D. J. de Waal R. M. (2001). Lack of lymphangiogenesis despite coexpression of VEGF-C and its receptor Flt-4 in uveal melanoma. Invest. Ophthalmol. Vis. Sci. 42 (7), 1422–1428. 11381041

[B25] Collin H. B. (1966). Endothelial cell lined lymphatics in the vascularized rabbit cornea. Invest. Ophthalmol. 5 (4), 337–354. 5912539

[B26] Conrady C. D. Zheng M. Stone D. U. Carr D. J. J. (2012). CD8+ T cells suppress viral replication in the cornea but contribute to VEGF-C-induced lymphatic vessel genesis. J. Immunol. 189 (1), 425–432. 10.4049/jimmunol.1200063 22649204 PMC3382000

[B27] Costache M. Patrascu O. M. Adrian D. Costache D. Sajin M. Ungureanu E. (2013). Ciliary body melanoma - a particularly rare type of ocular tumor. Case report and general considerations. Maedica (Bucur). 8 (4), 360–364. 24790669 PMC3968473

[B28] Cursiefen C. Chen L. Borges L. P. Jackson D. Cao J. Radziejewski C. (2004). VEGF-A stimulates lymphangiogenesis and hemangiogenesis in inflammatory neovascularization via macrophage recruitment. J. Clin. Invest. 113 (7), 1040–1050. 10.1172/JCI20465 15057311 PMC379325

[B29] Cursiefen C. Maruyama K. Bock F. Saban D. Sadrai Z. Lawler J. (2011). Thrombospondin 1 inhibits inflammatory lymphangiogenesis by CD36 ligation on monocytes. J. Exp. Med. 208 (5), 1083–1092. 10.1084/jem.20092277 21536744 PMC3092349

[B30] Cursiefen C. Maruyama K. Jackson D. G. Streilein J. W. Kruse F. E. (2006b). Time course of angiogenesis and lymphangiogenesis after brief corneal inflammation. Cornea 25 (4), 443–447. 10.1097/01.ico.0000183485.85636.ff 16670483

[B31] Cursiefen C. Rummelt C. Jünemann A. Vorwerk C. Neuhuber W. Kruse F. E. (2006a). Absence of blood and lymphatic vessels in the developing human cornea. Cornea 25 (6), 722–726. 10.1097/01.ico.0000214230.21238.3d 17077668

[B32] Dahlmann-Noor A. Muthusamy K. Hingorani M. (2021). Severe allergic eye disease: what to do and when? Clin. Exp. Allergy 51 (8), 989–991. 10.1111/cea.13985 34337807

[B33] DelMonte D. W. Kim T. (2011). Anatomy and physiology of the cornea. J. Cataract. Refract Surg. 37 (3), 588–598. 10.1016/j.jcrs.2010.12.037 21333881

[B34] Detry B. Blacher S. Erpicum C. Paupert J. Maertens L. Maillard C. (2013). Sunitinib inhibits inflammatory corneal lymphangiogenesis. Invest. Ophthalmol. Vis. Sci. 54 (5), 3082–3093. 10.1167/iovs.12-10856 23580490

[B35] Dietrich T. Bock F. Yuen D. Hos D. Bachmann B. O. Zahn G. (2010). Cutting edge: lymphatic vessels, not blood vessels, primarily mediate immune rejections after transplantation. J. Immunol. 184 (2), 535–539. 10.4049/jimmunol.0903180 20018627 PMC4725297

[B36] Doh S. J. Yamakawa M. Santosa S. M. Montana M. Guo K. Sauer J. R. (2018). Fluorescent reporter transgenic mice for *in vivo* live imaging of angiogenesis and lymphangiogenesis. Angiogenesis 21 (4), 677–698. 10.1007/s10456-018-9629-2 29971641 PMC6472480

[B37] Dohlman T. H. Omoto M. Hua J. Stevenson W. Lee S. M. Chauhan S. K. (2015). VEGF-trap aflibercept significantly improves long-term graft survival in high-risk corneal transplantation. Transplantation 99 (4), 678–686. 10.1097/TP.0000000000000512 25606789

[B38] Dong Y. Kase S. Dong Z. Fukuhara J. Tagawa Y. Ishizuka E. T. (2016). Regulation of vascular endothelial growth factor-C by tumor necrosis factor-α in the conjunctiva and pterygium. Int. J. Mol. Med. 38 (2), 545–550. 10.3892/ijmm.2016.2647 27314284

[B39] Ecoiffier T. Yuen D. Chen L. (2010). Differential distribution of blood and lymphatic vessels in the murine cornea. Invest. Ophthalmol. Vis. Sci. 51 (5), 2436–2440. 10.1167/iovs.09-4505 20019372 PMC2868485

[B40] Farooq A. V. Shukla D. (2012). Herpes simplex epithelial and stromal keratitis: an epidemiologic update. Surv. Ophthalmol. 57 (5), 448–462. 10.1016/j.survophthal.2012.01.005 22542912 PMC3652623

[B41] Ferrari G. Dastjerdi M. H. Okanobo A. Cheng S. F. Amparo F. Nallasamy N. (2013). Topical ranibizumab as a treatment of corneal neovascularization. Cornea 32 (7), 992–997. 10.1097/ICO.0b013e3182775f8d 23407316 PMC3920979

[B42] Flynn T. H. Ohbayashi M. Dawson M. Larkin D. F. P. Ono S. J. (2011). The effect of perioperative allergic conjunctivitis on corneal lymphangiogenesis after corneal transplantation. Br. J. Ophthalmol. 95 (10), 1451–1456. 10.1136/bjo.2010.201939 21653212

[B43] François M. Caprini A. Hosking B. Orsenigo F. Wilhelm D. Browne C. (2008). Sox18 induces development of the lymphatic vasculature in mice. Nature 456 (7222), 643–647. 10.1038/nature07391 18931657

[B44] Fukuhara J. Kase S. Ohashi T. Ando R. Dong Z. Noda K. (2013). Expression of vascular endothelial growth factor C in human pterygium. Histochem Cell Biol. 139 (2), 381–389. 10.1007/s00418-012-1019-z 22910845

[B45] Fukumoto A. Maruyama K. Walsh T. Kajiya K. Hamuro J. D’Amore P. A. (2010). Intracellular thiol redox status regulates lymphangiogenesis and dictates corneal limbal graft survival. Invest. Ophthalmol. Vis. Sci. 51 (5), 2450–2458. 10.1167/iovs.09-4618 20042658

[B46] Gale N. W. Thurston G. Hackett S. F. Renard R. Wang Q. McClain J. (2002). Angiopoietin-2 is required for postnatal angiogenesis and lymphatic patterning, and only the latter role is rescued by angiopoietin-1. Dev. Cell 3 (3), 411–423. 10.1016/s1534-5807(02)00217-4 12361603

[B47] Gao N. Liu X. Wu J. Li J. Dong C. Wu X. (2017). CXCL10 suppression of hem- and lymph-angiogenesis in inflamed corneas through MMP13. Angiogenesis 20 (4), 505–518. 10.1007/s10456-017-9561-x 28623423 PMC5702464

[B48] Goyal S. Chauhan S. K. Dana R. (2012). Blockade of prolymphangiogenic vascular endothelial growth factor C in dry eye disease. Arch. Ophthalmol. 130 (1), 84–89. 10.1001/archophthalmol.2011.266 21911653 PMC3629840

[B49] Goyal S. Chauhan S. K. El Annan J. Nallasamy N. Zhang Q. Dana R. (2010). Evidence of corneal lymphangiogenesis in dry eye disease: a potential link to adaptive immunity? Arch. Ophthalmol. 128 (7), 819–824. 10.1001/archophthalmol.2010.124 20625040 PMC3089983

[B50] Gurung H. R. Carr M. M. Bryant K. Chucair-Elliott A. J. Carr D. J. (2018). Fibroblast growth factor-2 drives and maintains progressive corneal neovascularization following HSV-1 infection. Mucosal Immunol. 11 (1), 172–185. 10.1038/mi.2017.26 28378806 PMC5628112

[B51] Heindl L. M. Hofmann T. N. Adler W. Knorr H. L. J. Holbach L. M. Naumann G. O. H. (2010b). Intraocular tumor-associated lymphangiogenesis a novel prognostic factor for ciliary body melanomas with extraocular extension? Ophthalmology 117 (2), 334–342. 10.1016/j.ophtha.2009.06.057 19892405

[B52] Heindl L. M. Hofmann T. N. Knorr H. L. J. Rummelt C. Schrödl F. Schlötzer-Schrehardt U. (2009). Intraocular lymphangiogenesis in malignant melanomas of the ciliary body with extraocular extension. Invest. Ophthalmol. Vis. Sci. 50 (5), 1988–1995. 10.1167/iovs.08-2935 19151383

[B53] Heindl L. M. Hofmann-Rummelt C. Adler W. Holbach L. M. Naumann G. O. H. Kruse F. E. (2010a). Tumor-associated lymphangiogenesis in the development of conjunctival squamous cell carcinoma. Ophthalmology 117 (4), 649–658. 10.1016/j.ophtha.2010.01.032 20346821

[B54] Hong Y. K. Detmar M. (2003). Prox1, master regulator of the lymphatic vasculature phenotype. Cell Tissue Res. 314 (1), 85–92. 10.1007/s00441-003-0747-8 12883994

[B55] Hos D. Bock F. Dietrich T. Onderka J. Kruse F. E. Thierauch K. H. (2008). Inflammatory corneal (lymph)angiogenesis is blocked by VEGFR-tyrosine kinase inhibitor ZK 261991, resulting in improved graft survival after corneal transplantation. Invest. Ophthalmol. Vis. Sci. 49 (5), 1836–1842. 10.1167/iovs.07-1314 18436817

[B56] Hos D. Bukowiecki A. Horstmann J. Bock F. Bucher F. Heindl L. M. (2017). Transient ingrowth of lymphatic vessels into the physiologically avascular cornea regulates corneal edema and transparency. Sci. Rep. 7 (1), 7227. 10.1038/s41598-017-07806-4 28775329 PMC5543160

[B57] Hos D. Matthaei M. Bock F. Maruyama K. Notara M. Clahsen T. (2019). Immune reactions after modern lamellar (DALK, DSAEK, DMEK) versus conventional penetrating corneal transplantation. Prog. Retin. Eye Res. 73, 100768. 10.1016/j.preteyeres.2019.07.001 31279005

[B58] Hos D. Regenfuss B. Bock F. Onderka J. Cursiefen C. (2011). Blockade of insulin receptor substrate-1 inhibits corneal lymphangiogenesis. Invest. Ophthalmol. Vis. Sci. 52 (8), 5778–5785. 10.1167/iovs.10-6816 21666240

[B59] Hou Y. Bock F. Hos D. Cursiefen C. (2021). Lymphatic trafficking in the eye: modulation of lymphatic trafficking to promote corneal transplant survival. Cells 10 (7), 1661. 10.3390/cells10071661 34359831 PMC8306557

[B60] Hou Y. Le V. N. H. Clahsen T. Schneider A. C. Bock F. Cursiefen C. (2017). Photodynamic therapy leads to time-dependent regression of pathologic corneal (lymph) angiogenesis and promotes high-risk corneal allograft survival. Investigative Ophthalmol. Vis. Sci. 58 (13), 5862–5869. 10.1167/iovs.17-22904 29145577

[B61] Hou Y. Le V. N. H. Tóth G. Siebelmann S. Horstmann J. Gabriel T. (2018). UV light crosslinking regresses mature corneal blood and lymphatic vessels and promotes subsequent high-risk corneal transplant survival. Am. J. Transpl. 18 (12), 2873–2884. 10.1111/ajt.14874 PMC6282984 29673063

[B62] Ikeda Y. Yonemitsu Y. Onimaru M. Nakano T. Miyazaki M. ichiro K. R. (2006). The regulation of vascular endothelial growth factors (VEGF-A, -C, and -D) expression in the retinal pigment epithelium. Exp. Eye Res. 83 (5), 1031–1040. 10.1016/j.exer.2006.05.007 16842779

[B63] Iwamoto T. Smelser G. K. (1965). Electron microscope studies on the mast cells and blood and lymphatic capillaries of the human corneal limbus. Invest. Ophthalmol. 4 (5), 815–834. 5831990

[B64] Jayaram H. Kolko M. Friedman D. S. Gazzard G. (2023). Glaucoma: now and beyond. Lancet 402, 1788–1801. 10.1016/s0140-6736(23)01289-8 37742700

[B65] Johnson M. McLaren J. W. Overby D. R. (2017). Unconventional aqueous humor outflow: a review. Exp. Eye Res. 158, 94–111. 10.1016/j.exer.2016.01.017 26850315 PMC4970980

[B66] Johnson N. C. Dillard M. E. Baluk P. McDonald D. M. Harvey N. L. Frase S. L. (2008). Lymphatic endothelial cell identity is reversible and its maintenance requires Prox1 activity. Genes Dev. 22 (23), 3282–3291. 10.1101/gad.1727208 19056883 PMC2600759

[B67] Kang G. J. Truong T. Huang E. Su V. Ge S. Chen L. (2016). Integrin alpha 9 blockade suppresses lymphatic valve formation and promotes transplant survival. Invest. Ophthalmol. Vis. Sci. 57 (14), 5935–5939. 10.1167/iovs.16-20130 27806381 PMC5096415

[B68] Kang H. Feng J. Peng Y. Liu Y. Yang Y. Wu Y. (2023). Human mesenchymal stem cells derived from adipose tissue showed a more robust effect than those from the umbilical cord in promoting corneal graft survival by suppressing lymphangiogenesis. Stem Cell Res. Ther. 14, 328. 10.1186/s13287-023-03559-2 37957770 PMC10644560

[B69] Kaplan H. J. (2007). Anatomy and function of the eye. Chem. Immunol. Allergy 92, 4–10. 10.1159/000099236 17264478

[B70] Katsuta H. Fukushima Y. Maruyama K. Hirashima M. Nishida K. Nishikawa S. I. (2013). EphrinB2-EphB4 signals regulate formation and maintenance of funnel-shaped valves in corneal lymphatic capillaries. Invest. Ophthalmol. Vis. Sci. 54 (6), 4102–4108. 10.1167/iovs.12-11436 23696610

[B71] Khan A. M. Kagan D. B. Gupta N. Navajas E. V. Jin Y. P. Yücel Y. H. (2013). Ciliary body lymphangiogenesis in uveal melanoma with and without extraocular extension. Ophthalmology 120 (2), 306–310. 10.1016/j.ophtha.2012.07.064 23062649

[B72] Kim J. Park D. Y. Bae H. Park D. Y. Kim D. Lee C. K. (2017). Impaired angiopoietin/Tie2 signaling compromises Schlemm’s canal integrity and induces glaucoma. J. Clin. Invest. 127 (10), 3877–3896. 10.1172/JCI94668 28920924 PMC5617682

[B73] Krishna U. Ajanaku D. Denniston A. K. Gkika T. (2017). Uveitis: a sight-threatening disease which can impact all systems. Postgrad. Med. J. 93 (1106), 766–773. 10.1136/postgradmedj-2017-134891 28942431

[B74] Kubo H. Cao R. Brakenhielm E. Mäkinen T. Cao Y. Alitalo K. (2002). Blockade of vascular endothelial growth factor receptor-3 signaling inhibits fibroblast growth factor-2-induced lymphangiogenesis in mouse cornea. Proc. Natl. Acad. Sci. U. S. A. 99 (13), 8868–8873. 10.1073/pnas.062040199 12070340 PMC124390

[B75] Kuonqui K. Campbell A. C. Sarker A. Roberts A. Pollack B. L. Park H. J. (2023). Dysregulation of lymphatic endothelial VEGFR3 signaling in disease. Cells 13 (1), 68. 10.3390/cells13010068 38201272 PMC10778007

[B76] Lan W. Petznick A. Heryati S. Rifada M. Tong L. (2012). Nuclear Factor-κB: central regulator in ocular surface inflammation and diseases. Ocul. Surf. 10 (3), 137–148. 10.1016/j.jtos.2012.04.001 22814642

[B77] Lee H. K. Lee S. M. Lee D. I. (2021). Corneal lymphangiogenesis: current pathophysiological understandings and its functional role in ocular surface disease. Int. J. Mol. Sci. 22 (21), 11628. 10.3390/ijms222111628 34769057 PMC8583961

[B78] Lee H. S. Chauhan S. K. Okanobo A. Nallasamy N. Dana R. (2011). Therapeutic efficacy of topical epigallocatechin gallate in murine dry eye. Cornea 30 (12), 1465–1472. 10.1097/ICO.0b013e31821c9b5a 21993466 PMC3703663

[B79] Lee H. S. Hos D. Blanco T. Bock F. Reyes N. J. Mathew R. (2015). Involvement of corneal lymphangiogenesis in a mouse model of allergic eye disease. Invest. Ophthalmol. Vis. Sci. 56 (5), 3140–3148. 10.1167/iovs.14-16186 26024097 PMC4451613

[B80] Li S. Shi S. Xia F. Luo B. Ha Y. Luisi J. (2022). CXCR3 deletion aggravates corneal neovascularization in a corneal alkali-burn model. Exp. Eye Res. 225, 109265. 10.1016/j.exer.2022.109265 36206861 PMC10191246

[B81] Lin H. Luo L. Ling S. Chen W. Liu Z. Zhong X. (2013). Lymphatic microvessel density as a predictive marker for the recurrence time of pterygium: a three-year follow-up study. Mol. Vis. 19, 166–173. 23378730 PMC3559093

[B82] Liu L. Ling S. Q. Li Q. L. Wang T. Ye H. Yang J. Z. (2012). Relations between lymphangiogenesis and the size of pterygium. Int. J. Ophthalmol. 5 (3), 312–316. 10.3980/j.issn.2222-3959.2012.03.12 22773979 PMC3388399

[B83] Liu Y. Shu Y. Yin L. Xie T. Zou J. Zhan P. (2021). Protective roles of the TIR/BB-loop mimetic AS-1 in alkali-induced corneal neovascularization by inhibiting ERK phosphorylation. Exp. Eye Res. 207, 108568. 10.1016/j.exer.2021.108568 33839112

[B84] Lou B. Wu W. Zeng L. Zhou W. Zhang X. Zhou X. (2022). Alleviating experimental allergic eye disease by inhibiting pro-lymphangiogenic VEGFR3 signal. Ocul. Surf. 26, 1–12. 10.1016/j.jtos.2022.07.002 35931408

[B85] Lyons O. Walker J. Seet C. Ikram M. Kuchta A. Arnold A. (2021). Mutations in EPHB4 cause human venous valve aplasia. JCI Insight 6 (18), e140952. 10.1172/jci.insight.140952 34403370 PMC8492339

[B86] Ma W. Gil H. J. Liu X. Diebold L. P. Morgan M. A. Oxendine-Burns M. J. (2021). Mitochondrial respiration controls the Prox1-Vegfr3 feedback loop during lymphatic endothelial cell fate specification and maintenance. Sci. Adv. 7 (18), eabe7359. 10.1126/sciadv.abe7359 33931446 PMC8087398

[B87] Mäkinen T. Veikkola T. Mustjoki S. Karpanen T. Catimel B. Nice E. C. (2001). Isolated lymphatic endothelial cells transduce growth, survival and migratory signals via the VEGF-C/D receptor VEGFR-3. EMBO J. 20 (17), 4762–4773. 10.1093/emboj/20.17.4762 11532940 PMC125596

[B88] Martín-López J. Pérez-Rico C. García-Honduvilla N. Buján J. Pascual G. (2019). Elevated blood/lymphatic vessel ratio in pterygium and its relationship with vascular endothelial growth factor (VEGF) distribution. Histol. Histopathol. 34 (8), 917–929. 10.14670/HH-18-095 30821336

[B89] Maruyama Y. Maruyama K. Kato Y. Kajiya K. Moritoh S. Yamamoto K. (2014). The effect of podoplanin inhibition on lymphangiogenesis under pathological conditions. Invest. Ophthalmol. Vis. Sci. 55 (8), 4813–4822. 10.1167/iovs.13-13711 24985477

[B90] Medawar P. B. (1948). Immunity to homologous grafted skin; the fate of skin homografts transplanted to the brain, to subcutaneous tissue, and to the anterior chamber of the eye. Br. J. Exp. Pathol. 29, 58–69. 18865105 PMC2073079

[B91] Messmer E. M. (2015). The pathophysiology, diagnosis, and treatment of dry eye disease. Dtsch. Arztebl Int. 112 (5), 71–81. quiz 82. 10.3238/arztebl.2015.0071 25686388 PMC4335585

[B92] Min J. H. Lee C. H. Ji Y. W. Yeo A. Noh H. Song I. (2016). Activation of dll4/notch signaling and hypoxia-inducible factor-1 alpha facilitates lymphangiogenesis in lacrimal glands in dry eye. PLoS One 11 (2), e0147846. 10.1371/journal.pone.0147846 26828208 PMC4734677

[B93] Nakao S. Zandi S. ichiro K. R. Sun D. Nakama T. Ishikawa K. (2013). Lack of lymphatics and lymph node-mediated immunity in choroidal neovascularization. Invest. Ophthalmol. Vis. Sci. 54 (6), 3830–3836. 10.1167/iovs.12-10341 23580489 PMC3671933

[B94] Narimatsu A. Hattori T. Koike N. Tajima K. Nakagawa H. Yamakawa N. (2019). Corneal lymphangiogenesis ameliorates corneal inflammation and edema in late stage of bacterial keratitis. Sci. Rep. 9, 2984. 10.1038/s41598-019-39876-x 30814667 PMC6393676

[B95] Niederkorn J. Y. (2006). See no evil, hear no evil, do no evil: the lessons of immune privilege. Nat. Immunol. 7 (4), 354–359. 10.1038/ni1328 16550198

[B96] Ocul Surf. (2007). The definition and classification of dry eye disease: report of the definition and classification subcommittee of the international dry eye WorkShop 2007. Ocul. Surf.;5(2). 75–92. 1 10.1016/s1542-0124(12)70081-2 17508116

[B97] Okanobo A. Chauhan S. K. Dastjerdi M. H. Kodati S. Dana R. (2012). Efficacy of topical blockade of interleukin-1 in experimental dry eye disease. Am. J. Ophthalmol. 154 (1), 63–71. 10.1016/j.ajo.2012.01.034 22541929 PMC3378826

[B98] Oliver G. Kipnis J. Randolph G. J. Harvey N. L. (2020). The lymphatic vasculature in the 21st century: novel functional roles in homeostasis and disease. Cell 182 (2), 270–296. 10.1016/j.cell.2020.06.039 32707093 PMC7392116

[B99] Özgürtaş T. Yücel Ç. Sertoğlu E. Hayran Y. Çolak S. Tekgöz E. (2022). Evaluation of the relationship of lymphangiogenesis markers with disease pathogenesis in patients with Behçet’s uveitis. Acta Clin. Belg. 77 (2), 387–395. 10.1080/17843286.2021.1890451 33629934

[B100] Pan Y. Wang W. Yago T. (2014). Transcriptional regulation of podoplanin expression by Prox1 in lymphatic endothelial cells. Microvasc. Res. 94, 96–102. 10.1016/j.mvr.2014.05.006 24944097

[B101] Park D. Y. Lee J. Park I. Choi D. Lee S. Song S. (2014). Lymphatic regulator PROX1 determines Schlemm’s canal integrity and identity. J. Clin. Invest. 124 (9), 3960–3974. 10.1172/JCI75392 25061877 PMC4153702

[B102] Patnam M. Dommaraju S. R. Masood F. Herbst P. Chang J. H. Hu W. Y. (2023). Lymphangiogenesis guidance mechanisms and therapeutic implications in pathological states of the cornea. Cells 12 (2), 319. 10.3390/cells12020319 36672254 PMC9856498

[B103] Qin Q. Hu K. He Z. Chen F. Zhang W. Liu Y. (2022). Resolvin D1 protects against Aspergillus fumigatus keratitis in diabetes by blocking the MAPK-NF-κB pathway. Exp. Eye Res. 216, 108941. 10.1016/j.exer.2022.108941 35077754

[B104] Ravichandran S. Natarajan R. (2022). Fine-needle diathermy for corneal vascularisation. Indian J. Ophthalmol. 70 (5), 1868. 10.4103/ijo.IJO_1013_22 PMC9332979 35502114

[B105] Refaian N. Schlereth S. L. Koch K. R. Notara M. Hos D. Mescher M. (2015). Comparing the hem- and lymphangiogenic profile of conjunctival and uveal melanoma cell lines. Invest. Ophthalmol. Vis. Sci. 56 (9), 5691–5697. 10.1167/iovs.15-16829 26313304

[B106] Regenfuss B. Bock F. Parthasarathy A. Cursiefen C. (2008). Corneal (lymph)angiogenesis--from bedside to bench and back: a tribute to Judah Folkman. Lymphat. Res. Biol. 6 (3–4), 191–201. 10.1089/lrb.2008.6348 19093792

[B107] Rho C. R. Choi J. S. Seo M. Lee S. K. Joo C. K. (2015). Inhibition of lymphangiogenesis and hemangiogenesis in corneal inflammation by subconjunctival Prox1 siRNA injection in rats. Invest. Ophthalmol. Vis. Sci. 56 (10), 5871–5879. 10.1167/iovs.14-14433 26348636

[B108] Rowe A. M. St Leger A. J. Jeon S. Dhaliwal D. K. Knickelbein J. E. Hendricks R. L. (2013). Herpes keratitis. Prog. Retin Eye Res. 32, 88–101. 10.1016/j.preteyeres.2012.08.002 22944008 PMC3529813

[B109] Sarkar A. Jayesh Sodha S. Junnuthula V. Kolimi P. Dyawanapelly S. (2022). Novel and investigational therapies for wet and dry age-related macular degeneration. Drug Discov. Today 27 (8), 2322–2332. 10.1016/j.drudis.2022.04.013 35460893

[B110] Schlereth S. L. Iden S. Mescher M. Ksander B. R. Bosch J. J. Cursiefen C. (2015). A novel model of metastatic conjunctival melanoma in immune-competent mice. Invest. Ophthalmol. Vis. Sci. 56 (10), 5965–5973. 10.1167/iovs.15-17290 26377082

[B111] Seo M. Choi J. S. Rho C. R. Joo C. K. Lee S. K. (2015). MicroRNA miR-466 inhibits Lymphangiogenesis by targeting prospero-related homeobox 1 in the alkali burn corneal injury model. J. Biomed. Sci. 22 (1), 3. 10.1186/s12929-014-0104-0 25573115 PMC4304626

[B112] Sertoglu E. Yücel Ç. Omma A. Hayran Y. Colak S. Sandıkçı S. C. (2022). Determination of serum vascular endothelial growth factor (VEGF) and VEGF receptor levels with VEGF gene polymorphisms in patients with Behçet’s uveitis. Adv. Clin. Exp. Med. 31 (3), 231–240. 10.17219/acem/143586 34918882 PMC13318138

[B113] Shen M. Yuan F. Jin J. Yuan Y. (2014). The effect of TC14012 on alkali burn-induced corneal neovascularization in mice. Ophthalmic Res. 52 (1), 17–24. 10.1159/000358201 24853648

[B114] Shi M. Zhang L. Ye E. A. Wang A. Li G. Chen L. (2020). Aqueous humor induces lymphatic regression on the ocular surface. Ocul. Surf. 18 (3), 505–510. 10.1016/j.jtos.2020.03.002 32173554 PMC8099855

[B115] Streilein J. W. (2003). Ocular immune privilege: therapeutic opportunities from an experiment of nature. Nat. Rev. Immunol. 3 (11), 879–889. 10.1038/nri1224 14668804

[B116] Subileau M. Acar N. Carret A. Bretillon L. Vilgrain I. Bailly S. (2020). Eye lymphatic defects induced by bone morphogenetic protein 9 deficiency have no functional consequences on intraocular pressure. Sci. Rep. 10 (1), 16040. 10.1038/s41598-020-71877-z 32994463 PMC7524742

[B117] Tam A. L. C. Gupta N. Zhang Z. Yücel Y. H. (2013). Latanoprost stimulates ocular lymphatic drainage: an *in vivo* nanotracer study. Transl. Vis. Sci. Technol. 2 (5), 3. 10.1167/tvst.2.5.3 PMC3763898 24049723

[B118] Tammela T. Alitalo K. (2010). Lymphangiogenesis: molecular mechanisms and future promise. Cell 140 (4), 460–476. 10.1016/j.cell.2010.01.045 20178740

[B119] Tang X. Sun J. Du L. Du H. Wang L. Mai J. (2016). Neuropilin-2 contributes to LPS-induced corneal inflammatory lymphangiogenesis. Exp. Eye Res. 143, 110–119. 10.1016/j.exer.2015.10.017 26500194

[B120] Tang X. Sun J. Wang X. Du L. Liu P. (2010). Blocking neuropilin-2 enhances corneal allograft survival by selectively inhibiting lymphangiogenesis on vascularized beds. Mol. Vis. 16, 2354–2361. 21139694 PMC2994732

[B121] Uhrin P. Zaujec J. Breuss J. M. Olcaydu D. Chrenek P. Stockinger H. (2010). Novel function for blood platelets and podoplanin in developmental separation of blood and lymphatic circulation. Blood 115 (19), 3997–4005. 10.1182/blood-2009-04-216069 20110424

[B122] Vaahtomeri K. Karaman S. Mäkinen T. Alitalo K. (2017). Lymphangiogenesis guidance by paracrine and pericellular factors. Genes Dev. 31 (16), 1615–1634. 10.1101/gad.303776.117 28947496 PMC5647933

[B123] van Beek J. G. M. van den Bosch Q. C. C. Naus N. Paridaens D. de Klein A. Kiliç E. (2019). Absence of intraocular lymphatic vessels in uveal melanomas with extrascleral growth. Cancers (Basel) 11 (2), 228. 10.3390/cancers11020228 30781402 PMC6406846

[B124] Vasalaki M. Fabian I. D. Reddy M. A. Cohen V. M. L. Sagoo M. S. (2017). Ocular oncology: advances in retinoblastoma, uveal melanoma and conjunctival melanoma. Br. Med. Bull. 121 (1), 107–119. 10.1093/bmb/ldw053 28069617

[B125] Wang D. Zhao Y. Zhou Y. Yang S. Xiao X. Feng L. (2023). Angiogenesis-an emerging role in organ fibrosis. Int. J. Mol. Sci. 24 (18), 14123. 10.3390/ijms241814123 37762426 PMC10532049

[B126] Wang L. Wang R. Xu C. Zhou H. (2020). Pathogenesis of herpes stromal keratitis: immune inflammatory response mediated by inflammatory regulators. Front. Immunol. 11, 766. 10.3389/fimmu.2020.00766 32477330 PMC7237736

[B127] Wang T. Li W. Cheng H. Zhong L. Deng J. Ling S. (2021). The important role of the chemokine Axis CCR7-CCL19 and CCR7-CCL21 in the pathophysiology of the immuno-inflammatory response in dry eye disease. Ocul. Immunol. Inflamm. 29 (2), 266–277. 10.1080/09273948.2019.1674891 31702421

[B128] Weinreb R. N. Aung T. Medeiros F. A. (2014). The pathophysiology and treatment of glaucoma: a review. JAMA 311 (18), 1901–1911. 10.1001/jama.2014.3192 24825645 PMC4523637

[B129] Wuest T. R. Carr D. J. J. (2010). VEGF-A expression by HSV-1-infected cells drives corneal lymphangiogenesis. J. Exp. Med. 207 (1), 101–115. 10.1084/jem.20091385 20026662 PMC2812544

[B130] Yamada J. Dana M. R. Sotozono C. Kinoshita S. (2003). Local suppression of IL-1 by receptor antagonist in the rat model of corneal alkali injury. Exp. Eye Res. 76 (2), 161–167. 10.1016/s0014-4835(02)00293-2 12565803

[B131] Yan H. Qi C. Ling S. Li W. Liang L. (2010). Lymphatic vessels correlate closely with inflammation index in alkali burned cornea. Curr. Eye Res. 35 (8), 685–697. 10.3109/02713681003793136 20673045

[B132] Yan Z. X. Jiang Z. H. Liu N. F. (2012). Angiopoietin-2 promotes inflammatory lymphangiogenesis and its effect can be blocked by the specific inhibitor L1-10. Am. J. Physiol. Heart Circ. Physiol. 302 (1), H215–H223. 10.1152/ajpheart.00895.2011 22058148

[B133] Yu J. Li Y. Li Z. Li H. Chen Y. Chen X. (2021). Subconjunctival injections of dimethyl fumarate inhibit lymphangiogenesis and allograft rejection in the rat cornea. Int. Immunopharmacol. 96, 107580. 10.1016/j.intimp.2021.107580 33823430

[B134] Yu T. Forrester J. V. Graham G. J. Kuffova L. (2018). The atypical chemokine receptor-2 does not alter corneal graft survival but regulates early stage of corneal graft-induced lymphangiogenesis. Graefes Arch. Clin. Exp. Ophthalmol. 256 (10), 1875–1882. 10.1007/s00417-018-4070-1 30054731 PMC6153595

[B135] Yu T. Schuette F. Christofi M. Forrester J. V. Graham G. J. Kuffova L. (2022). The atypical chemokine receptor-2 fine-tunes the immune response in herpes stromal keratitis. Front. Immunol. 13, 1054260. 10.3389/fimmu.2022.1054260 36518752 PMC9742518

[B136] Yücel Y. H. Johnston M. G. Ly T. Patel M. Drake B. Gümüş E. (2009). Identification of lymphatics in the ciliary body of the human eye: a novel “uveolymphatic” outflow pathway. Exp. Eye Res. 89 (5), 810–819. 10.1016/j.exer.2009.08.010 19729007

[B137] Yuen D. Grimaldo S. Sessa R. Ecoiffier T. Truong T. Huang E. (2014). Role of angiopoietin-2 in corneal lymphangiogenesis. Invest. Ophthalmol. Vis. Sci. 55 (5), 3320–3327. 10.1167/iovs.13-13779 24781940 PMC4039380

[B138] Yun H. Yee M. B. Lathrop K. L. Kinchington P. R. Hendricks R. L. St Leger A. J. (2020). Production of the cytokine VEGF-A by CD4+ T and myeloid cells disrupts the corneal nerve landscape and promotes herpes stromal keratitis. Immunity 53 (5), 1050–1062. 10.1016/j.immuni.2020.10.013 33207210 PMC7682749

[B139] Zarrin A. A. Bao K. Lupardus P. Vucic D. (2021). Kinase inhibition in autoimmunity and inflammation. Nat. Rev. Drug Discov. 20 (1), 39–63. 10.1038/s41573-020-0082-8 33077936 PMC7569567

[B140] Zhang L. Li G. Sessa R. Kang G. J. Shi M. Ge S. (2017). Angiopoietin-2 blockade promotes survival of corneal transplants. Invest. Ophthalmol. Vis. Sci. 58 (1), 79–86. 10.1167/iovs.16-20485 28061513 PMC5231909

[B141] Zhang W. Schönberg A. Bassett F. Hadrian K. Hos D. Becker M. (2022b). Different murine high-risk corneal transplant settings vary significantly in their (Lymph)angiogenic and inflammatory cell signatures. Invest. Ophthalmol. Vis. Sci. 63 (13), 18. 10.1167/iovs.63.13.18 PMC9769342 36534386

[B142] Zhang W. Schönberg A. Hamdorf M. Georgiev T. Cursiefen C. Bock F. (2022a). Preincubation of donor tissue with a VEGF cytokine trap promotes subsequent high-risk corneal transplant survival. Br. J. Ophthalmol. 106 (11), 1617–1626. 10.1136/bjophthalmol-2021-319745 34810177

[B143] Zhu J. Inomata T. Fujimoto K. Uchida K. Fujio K. Nagino K. (2021). *Ex vivo*-Induced bone marrow-derived myeloid suppressor cells prevent corneal allograft rejection in mice. Invest. Ophthalmol. Vis. Sci. 62 (7), 3. 10.1167/iovs.62.7.3 PMC8185403 34061951

[B144] Zhuo W. Jia L. Song N. Lu X. A. Ding Y. Wang X. (2012). The CXCL12-CXCR4 chemokine pathway: a novel axis regulates lymphangiogenesis. Clin. Cancer Res. 18 (19), 5387–5398. 10.1158/1078-0432.CCR-12-0708 22932666

[B145] Zimmermann P. Dietrich T. Bock F. Horn F. K. Hofmann-Rummelt C. Kruse F. E. (2009). Tumour-associated lymphangiogenesis in conjunctival malignant melanoma. Br. J. Ophthalmol. 93 (11), 1529–1534. 10.1136/bjo.2008.147355 19628489 PMC2760727

